# The Multifunctional Role of Herbal Products in the Management of Diabetes and Obesity: A Comprehensive Review

**DOI:** 10.3390/molecules27051713

**Published:** 2022-03-06

**Authors:** Md. Mominur Rahman, Md. Rezaul Islam, Sheikh Shohag, Md. Emon Hossain, Md. Saidur Rahaman, Fahadul Islam, Muniruddin Ahmed, Saikat Mitra, Mayeen Uddin Khandaker, Abubakr M. Idris, Kumarappan Chidambaram, Talha Bin Emran, Simona Cavalu

**Affiliations:** 1 Department of Pharmacy, Faculty of Allied Health Sciences, Daffodil International University, Dhaka 1207, Bangladesh; mominur.ph@gmail.com (M.M.R.); md.rezaulislam100ds@gmail.com (M.R.I.); emonhossain281033@gmail.com (M.E.H.); mdsaidur569@gmail.com (M.S.R.); fahadulislamdiu@gmail.com (F.I.); drmuniruddin@gmail.com (M.A.); 2 Department of Biochemistry and Molecular Biology, Faculty of Life Science, Bangabandhu Sheikh Mujibur Rahman Science and Technology University, Gopalganj 8100, Bangladesh; sheikhshohag.bmb@gmail.com; 3 Department of Pharmacy, Faculty of Pharmacy, University of Dhaka, Dhaka 1000, Bangladesh; saikatmitradu@gmail.com; 4 Centre for Applied Physics and Radiation Technologies, School of Engineering and Technology, Sunway University, Bandar Sunway, Petaling Jaya 47500, Malaysia; mayeenk@sunway.edu.my; 5 Department of Chemistry, College of Science, King Khalid University, Abha 62529, Saudi Arabia; abubakridris@hotmail.com; 6 Research Center for Advanced Materials Science (RCAMS), King Khalid University, Abha 62529, Saudi Arabia; 7 Department of Pharmacology and Toxicology, College of Pharmacy, King Khalid University, Abha 62529, Saudi Arabia; kumarappan@kku.edu.sa; 8 Department of Pharmacy, BGC Trust University Bangladesh, Chittagong 4381, Bangladesh; 9 Faculty of Medicine and Pharmacy, University of Oradea, 410087 Oradea, Romania

**Keywords:** diabetes, obesity, herbal products, treatment, hyperglycemia

## Abstract

Obesity and diabetes are the most demanding health problems today, and their prevalence, as well as comorbidities, is on the rise all over the world. As time goes on, both are becoming big issues that have a big impact on people’s lives. Diabetes is a metabolic and endocrine illness set apart by hyperglycemia and glucose narrow-mindedness because of insulin opposition. Heftiness is a typical, complex, and developing overall wellbeing worry that has for quite some time been connected to significant medical issues in individuals, all things considered. Because of the wide variety and low adverse effects, herbal products are an important hotspot for drug development. Synthetic compounds are not structurally diverse and lack drug-likeness properties. Thus, it is basic to keep on exploring herbal products as possible wellsprings of novel drugs. We conducted this review of the literature by searching Scopus, Science Direct, Elsevier, PubMed, and Web of Science databases. From 1990 until October 2021, research reports, review articles, and original research articles in English are presented. It provides top to bottom data and an examination of plant-inferred compounds that might be utilized against heftiness or potentially hostile to diabetes treatments. Our expanded comprehension of the systems of activity of phytogenic compounds, as an extra examination, could prompt the advancement of remedial methodologies for metabolic diseases. In clinical trials, a huge number of these food kinds or restorative plants, as well as their bioactive compounds, have been shown to be beneficial in the treatment of obesity.

## 1. Introduction

Diabetes mellitus (DM) is a metabolic disorder that may be caused by hereditary or environmental causes, and it increases the risk of various diseases in patients. Currently, available therapies are effective for some individuals, but not all, and there are currently no effective medicines available to combat this illness. As a result, there is a high need for novel antidiabetic medications [1,2]. On the other hand, obesity is a significant public health concern that may lead to the development of a variety of other health problems, such as type-2 diabetes (T2DM). To the extent that allopathic medicines and homeopathic medicines are only partly effective in treating this health issue, new alternative methods must be developed with long-term efficacy and minimal side effects in mind [3]. Compared to manufactured molecules, herbal products have a greater degree of intrinsic structural variety. So far they have emerged as a vital source of bioactive molecules and have contributed significantly to the identification of novel drug-discovery leads [1]. Obesity is a complicated condition caused by a combination of genetic, nutritional, lifestyle, and environmental variables [4]. Obesity and overweight are on the rise all across the world. Diabetes and cardiovascular and locomotory illnesses are among the comorbidities associated with obesity. It also has a substantial impact on the individual’s social, financial, and psychological well-being, which may contribute to the onset of depression [5].

Many conventional medications are used to treat obesity in today’s world. The availability of these medications, as well as their potentially hazardous side effects, limits their use. As a result, developing safe, effective, and cost-effective entities with easy accessibility is critical. Plant-derived medications are thought to be the first line of defense in keeping people healthy by preventing diseases and their complications [6,7]. By acting on several targets, the anti-obesity impact of synergistic polyherbal formulations will be amplified. Furthermore, these herbs have numerous health benefits in addition to their anti-obesity properties [8]. As a result, using certain plants and their compounds may be a useful strategy for managing obesity and related diseases [9]. Regular synthetics are complex mixes or compounds found in nature that are present in living things [10]. Plants, animals, and microbes are all dependent on regular synthetics [11]. In this review, we look at common combinations in general, but we focus on plant-specific blends specifically. Regular synthetics have been a good source of new drugs for a long time. Around half of the prescriptions supported by the Food and Drug Administration (FDA) are phytogenic herbal products. Herbal products have played an important role in the development of pharmaceuticals [12,13]. In light of their variety, regular herbal products have been an amazing hotspot for producing new prescriptions. Herbal particles have this property which permits them to be blended into drugs with complex designs and organic intensity that contrast from other synthetic mixtures [14,15]. Regular synthetics are additionally utilized in the improvement of medications as the recognizable proof and investigation of illness targets and pathways [11].

Diabetes has been known since old times, and the principal side effects were inordinate thirst, ceaseless peeing, and sleepiness. Heftiness is a critical danger factor for a scope of ongoing diseases, including T2DM, which is related to more noteworthy clinical consideration costs and a more restricted future. Free fatty acids (FFA) have a strong link to obesity, discomfort, and insulin resistance; therefore, lowering prolonged plasma levels is critical. In the therapy of obesity and T2DM, FFA should be a critical healing aim. As indicated by the World Health Organization (WHO), 35% of individuals possessed around 20 pounds of overabundant weight in 2008, with 11% being corpulent. Besides, the pervasiveness of T2DM has expanded from under 10% in 1980 to over 30% in recent years [16,17]. Glucose-lowering medicines incorporate insulin sensitizers (biguanides, metformin, and thiazolidinediones), insulin secretagogues (sulfonylureas, meglitinides), and glucosidase inhibitors (acarbose, miglitol) [18]. Most glucose-lowering treatments can cause genuine hypoglycemia, causing liver cell harm, lactic acidosis, irreversible neurological need, stomach-related pain, cerebral agony, and shakiness [19,20,21]. Therefore, researchers are attempting to foster more viable medications with fewer antagonistic impacts. Drug disclosure from restorative plants has yielded promising results against an arrangement of pharmacological targets, including T2DM and power. Customary things and dynamic particles obtained from them may be sensible choices for treating T2DM and related results with insignificant accidental impacts. A wide scope of solid restorative plants and their regular bioactive blends have been effectively shown to have antidiabetic abilities [22]. Since old times, various recuperating plants have been used to fix and forestall diabetes and related confusions [23,24].

In this review, we focused on the relation between diabetes and obesity, which is a burning issue of the current world, and the medications of diabetes and obesity from plant-derived herbal products concentrating on various therapeutic targets.

## 2. Pathogenesis of Obesity

**Figure 1:**
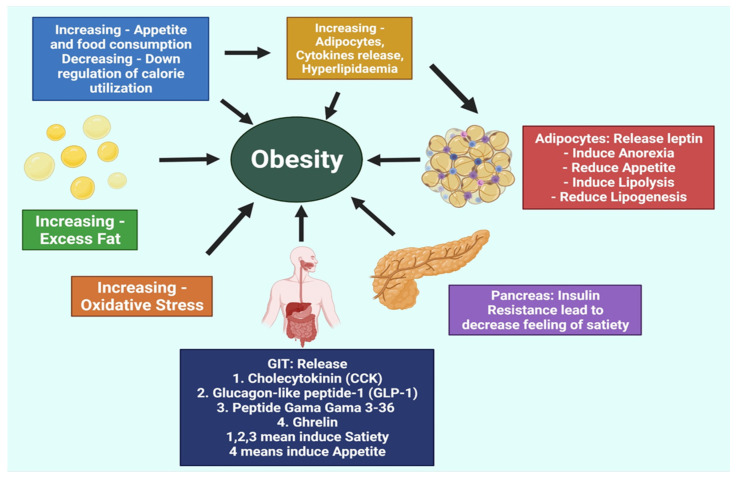
The fundamental model of obesity’s etiology. GIT, gastrointestinal tract.

The most basic cause of obesity is either increased hunger or decreased calorie consumption due to controlling cellular functioning, physical activity, and other aspects of a person’s life (Figure 1). The accumulation of excess adipocytes leads to increased cytokine production, resulting in the development of vascular tissues. Hyperlipidemia, cardiovascular irregularities, and atherosclerosis all go hand in hand when it comes to consequences. Atherosclerosis contributes to obesity because obese people are at an increased risk of numerous ailments, such as colorectal cancer, gallstones, liver and gut disease, etc. Because of this, managing obesity is a highly effective strategy for preventing and dealing with these co-morbidities. Reducing appetite or increasing calorie expenditure can help to control weight. Hormones and receptors responsible for hunger and satiety regulate the appetite. Reduced sitting time can also reduce white adipose tissue buildup. By using these measures, people can assist with preventing obesity and its repercussions [25].

The secretion of fatty acids and triglycerides in the circulation results in the formation of fat cells all over the body, followed by atherosclerosis. These metabolic disorders may also arise as a result of this phenomenon. For the management of obesity, we must reduce circulation and stored fat levels. Additionally, as oxidative stress is a frequent element in some pathological disorders, such as obesity and other conditions, reducing oxidative stress can help to mitigate the susceptible repercussions of obesity and other difficulties. In addition to secreting the three substances, adipocytes secrete an adipocytokine, also known as lectin, adiponectin, and visfatin. Autophagy can be induced by adiponectin in the breast, colon, prostate, and female-specific carcinogenesis [26,27,28]. Therefore, adiponectin suppression would be helpful in the prevention of obesity-related carcinogenesis. Hormones such as epinephrine, norepinephrine, and cortisol increase the release of insulin, which regulates blood glucose levels and controls body fat. As a result, any single part of this basic physiological sequence is unbalanced, which causes obesity. Insulin has an important role in managing obesity and is associated with diabetes [4,25].

Dopamine regulates fat and helps the pancreas and the digestive tract to release their respective hormones. Hormones that sustain hunger, satiety, and body fat are under homeostatic control, but hormone abnormalities can lead to obesity. This means that in the development of novel anti-obesity drugs, these elements and their impact are extremely significant [4,9,29].

## 3. Obesity and Diabetes

Obesity and diabetes, despite their differences in symptoms, share certain common characteristics. Insulin resistance has an impact on a huge number of persons [30]. Insulin resistance is a term used to describe diabetes. Many diabetic individuals are overweight or have an excess of stomach fat, but not all. On account of these shared characteristics, drug treatments that address both heftiness and diabetes are required to be created. Regular synthetic compounds that sway corpulence or diabetes have been found in various investigations. Ordinary synthetic materials have quite recently been used in several cases [31].

### 3.1. Obesity: Current Concerns and Treatments

Obesity is becoming all the more a worldwide issue, especially in developed nations. The WHO characterizes heftiness as a body mass index (BMI) of an excess of 30 kg/m^2^ [32]. A weight record of under 25 kg/m^2^ is viewed as typical, while a weight record of 25 to 30 kg/m^2^ is viewed as overweight. Hypertension, T2DM, hyperlipidemia, and coronary supply route illness are a couple of the drawn-out impacts of abundant weight. The following four are the most well-known reasons for corpulence: food/drink admittance, activity (workout), inherited elements [33], and clinical issues [34]. Food and drink provide calorie information, whereas vigorous work provides calorie surges. Caloric balance is achieved by combining these elements in the right proportions. Heftiness is caused by a sedentary lifestyle, excessive food consumption, and the consumption of deplorable food variety that disrupts the caloric balance. Digestion has an impact on stoutness as well. Heavy people’s metabolic rates are below that typical for healthy people. At the point when stout individuals shed pounds, their low metabolic rate does not change [35]. Accordingly, people who were previously overweight should be mindful of their food consumption since they can quickly recover their weight. Hefty individuals’ metabolic rates are below normal compared to lighter people. At the point when corpulent individuals get thinner, their low metabolic rate does not change [32]. Thus, people who were once overweight should be careful about their food consumption since they can quickly regain the weight.

Obesity can be addressed in a variety of ways. Smothering hunger is one method for reducing food intake [36]. A variety of factors, including neurological and hormonal signals, influence hunger control. Peptides and synthetics in the central and peripheral coordinate food affirmation [37]. Food affirmation increases when an orexigenic sign, for example, Neuropeptide Y (NPY), Agouti-related peptide (AgRP), orexin, or ghrelin, is generated. At the point when an anorexigenic sign is requested, in any case, food utilization diminishes. Insulin, leptin, peptide YY3–36, obestatin, cholecystokinin (CCK), glucagon-like peptide (GLP), and serotonin are, on the whole, anorexigenic engineered mixtures and peptides. Various drug organizations have tried different methods by utilizing a mix of these engineered synthetic compounds and neural contributions to treating burliness. FDA-endorsed drugs for obesity incorporate the gastric and pancreatic lipase inhibitor orlistat (Xenical), the endocannabinoid receptor blocker rimonabant (Acomplia), and the monoamine-reuptake inhibitor sibutramine (Reductil) [38].

### 3.2. Diabetes: Current Concerns and Treatments

Hyperglycemia, which is brought about by an absence of insulin discharge as insulin advancement, is an indication of diabetes [39]. Cardiovascular tainting, retinopathy, neuropathy, nephropathy, and diabetic foot affliction could all be treated with hyperglycemia movement [40]. Diabetes can be achieved by a variety of factors. Autoimmunity, for example, can hurt pancreatic cells, achieving insulin deficiency. Insulin resistance can be achieved by bizarre insulin affirmation. Along these lines, signal transduction in insulin flagging is weakened, bringing about hyperglycemia. Numerous diabetic individuals have both of these physiological defects. Be that as it may, the essential etiology of hyperglycemia remains uncertain. Diabetes is divided into several types, with type 1 and type 2 being the most common. Type 1 diabetes (T1DM) is caused by the nutrition system’s destruction of cells, which results in a lack of insulin production. Only roughly 5–10% of diabetics are affected by T1DM. Insulin obstruction and powerlessness to react suitably to hyperglycemia cause T2DM. T2DM influences by far most diabetics (90–95%). A few diabetics can handle their blood glucose levels with exercise, a smart dieting design, and an oral glucose-lowering remedy, contingent upon their condition. These diabetic patients do not need insulin from an outside source to live. Those with serious cell harm, then again, cannot handle their blood glucose levels by fundamental action and sustenance. They need consistent insulin imbuements to remain alive [39]. T2DM treatment is constantly in development. Metformin [41] is the most regularly utilized prescription for T2DM. Metformin diminishes hepatic glucose creation and plasma insulin levels while likewise further developing insulin affectability in patients with fringe issues, considering expanded glucose maintenance [42]. Metformin is a hypoglycemic medication that helps T2DM patients live longer. Metformin was originally developed from herbal compounds found in the plant *Galega officinalis*. Metformin has various incidental effects, including sickness, swelling, and flatulating. Other diabetes medications have been developed, yet none are as successful or convincing as metformin [41]. Adjusting one’s way of life is another type of diabetes treatment. In some situations, lifestyle adjustments are preferable to medications. As part of a way-of-life intervention, a low-calorie, low-fat eating regimen is combined with at least 150 min of respectably concentrated exercise per week [43]. This proposes that phytogenic substances, as opposed to synthetically produced prescriptions, are possibly more appropriate for diabetic treatment.

**Figure 2:**
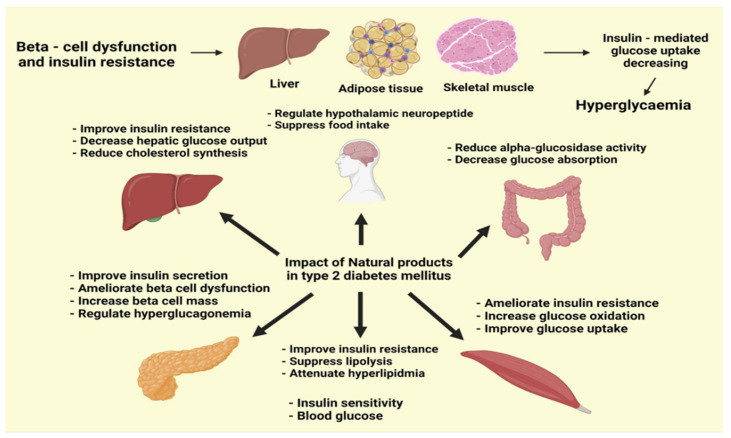
Natural products in type 2 diabetes mellitus.

DM is expected to become a pandemic worldwide, accompanied by metabolic and endocrine diseases, according to epidemiological studies. T2DM affects the majority of diabetic individuals, causing insulin resistance and insulin secretion problems. Dietary control, moderate exercise, and hypoglycemic and lipid-lowering medications are the most common treatments for T2DM. Despite the therapeutic benefits of most medicines for the treatment of T2DM, the majority of them might cause unwanted side effects. Herbal products have emerged as important sources of bioactive molecules for anti-T2DM medication development, given the pathophysiology of T2DM (Figure 2). Recently, a growing number of herbal products have been shown to exhibit anti-T2DM effects, prompting extensive research into the likely mechanisms [44].

## 4. Relationship between Diabetes and Obesity

Many studies have tracked down a significant connection between stoutness and the advancement of diabetes. Individuals who are overweight, especially around the stomach, are more insulin safe [45,46,47,48] and may battle to keep up with great diabetes control [49,50,51]. The expanded production of adipokines or cytokines, for example, tumor putrefaction factor, resistin, and retinol-restricting protein 4 [52] have been proposed to connect stoutness with insulin obstruction, which advances to diabetes. An overabundance of muscle versus fat, especially instinctive fat, delivers more FFAs into the circulatory system. Expanded FFA levels impact insulin flagging and lead the liver and skeletal muscles to move toward expanded FFA oxidation for energy creation while restraining chemicals in the glycolytic falls. Therefore, the liver and skeletal muscle cells’ capacity to acknowledge and process glucose decreases. Besides, the limit of tissues to store glucose as glycogen decreases, and cells gather a larger number of fatty substances as opposed to glycogen. Moreover, an Indian has a much greater muscle to fat ratio than a Caucasian with a comparative BMI and blood glucose level. An overabundance of muscle versus fat and low bulk have been proposed as clarifications for the high frequency of hyperinsulinemia and the high danger of T2DM in Asian Indians [53]. As BMI exceeds around 25 kg/m, the danger of diabetes rises dramatically [42]. A BMI of more than 23 was connected to an increased risk of T2DM in a huge cross-sectional investigation of moderately aged Indians [54]. Insulin opposition is supported by instinctive fat, which raises the danger of diabetes. Diabetic patients are frequently asked to exercise and lose weight. Weight, for quite a while, has unfortunate results on glucose homeostasis, such as expanded protection from glucose disposal and diminished insulin discharge. Stoutness is altogether connected to glucose removal obstruction, which brings about raised fasting and post-load blood insulin fixations. Heftiness for an all-inclusive timeframe could intensify this opposition [55]. Albeit the overabundance of fat in any part of the body is connected to an expanded danger of T2DM, it is generally acknowledged that an aggregation of stomach fat (‘local’ heftiness), as estimated by a higher midsection to-hip proportion, is an autonomous danger factor for T2DM, paying little mind to weight seriousness [56]. Expanded intra-stomach (instinctive) corpulence is generally to a fault. Through systems of intracellular lipotoxicity, unnecessary lipids amassing in muscle and liver additionally increase the danger of T2DM.

### Genetic Factors Linking Obesity and Diabetes

Obesity and diabetes are instances of multifactorial sicknesses that arise because of the interchange of different hereditary and ecological factors. There is evidence that builds up the hereditary connection between fat and diabetes. Forty qualities have been connected to T2DM by a genome-wide association study (GWAS) and up-and-comer quality methodologies and a comparative number of qualities have been connected to heftiness by GWAS and up-and-comer quality methodologies. Most T2DM qualities give off an impression of being connected to a B-cell glitch, with far fewer occupied with insulin obstruction pathways random to fat [57,58,59,60]. A superior comprehension of the hereditary qualities and organic movement of the B-cell can help specialists discover potential go-betweens that incline hefty individuals to T2DM as present new treatment targets. Notwithstanding the way that different diabetes and weight-related qualities have been found, it is accepted that the realized qualities can foresee 15% of T2DM and 5% of obesity hazards [61,62]. Various loci on chromosomes that influence heftiness-related characteristics have been found in ongoing genome-wide examinations [63]. It is conceivable that a portion of the affectability to T2DM and weight is owing to shared qualities. Five covering chromosomal areas for T2DM and heftiness have been recognized by contrasting all distributed genome checks for the two illnesses, and by examining these five vulnerability loci for T2DM and stoutness, 27 useful competitor qualities engaged with eating conduct, digestion, and aggravation have been pinpointed. These qualities could highlight an organic association between the two sicknesses [64]. At the point when the characterized heftiness pathways were contrasted with the characterized non-insulin-subordinate diabetes mellitus (NIDDM)- applicable pathways, it was found that the corpulence important pathways contain a quality set identified with the insulin receptor and that the NIDDM-pertinent quality set contains qualities that are 2-overlap up-managed by insulin. Besides that, the significant heftiness and NIDDM systems are totally extraordinary [65].

## 5. Phytogenic Compounds

**Figure 3:**
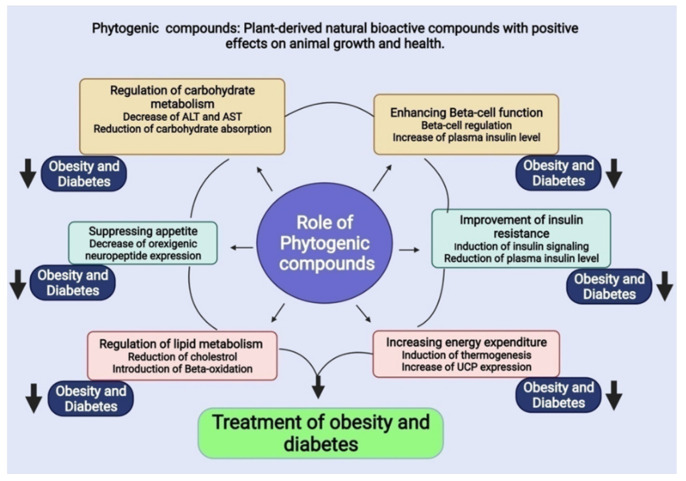
Phytogenic chemicals and diabetes. Phytogenic substances as possible options for obesity and diabetes therapies are shown.

Phytogenic compounds have been utilized to treat an extent of illnesses for a significantly long time [12,13]. For the treatment of stoutness and diabetes, for instance, a huge number of phytogenic blends have been investigated. Likewise, the phytogenic escalates analyzed in this survey could be significant in the treatment of weight and diabetes. The effects of phytogenic compounds are studied in more important significance in the spaces following and in Figure 3.

### 5.1. Possible Therapeutic Compounds for Obesity

#### 5.1.1. Compounds Suppress Food Intake

##### *Panax quinquefolius* (American Ginseng)

Ginsenoside is a foe of fat substances isolated from ginseng. Ginsenosides are copious in *Panax ginseng* (Asian ginseng) and *Panax quinquefolius* (American ginseng). On the glucose and fat processing frameworks, ginsenoside Rb1 has a scope of constructive outcomes. Ginsenoside Rb1 (10 mg/kg) was implanted intraperitoneally into a run-of-the-mill eating routine dealt with mice and high-fat unhealthy-eating supported mice packs for three weeks, and it reduced body weight, fat substance, serum leptin, and serum nitric oxide back to normally observed levels or lower than the normal group. Additionally, the paraventricular focus of the operational hub appeared to diminish orexigenic neuropeptide Y (NPY) expression while boosting anorexigenic CCK expression when the high-fat diet (HFD) pack was given ginsenoside Rb1. *P. ginseng* contains various ginsenosides, all of which help to diminish pancreatic lipase creation [66].

##### *Panax ginseng* (Asian Ginseng)

The proportions of ginsenosides Re, Rb2, and Rd in the result of *P. ginseng* are essentially higher in the root [67]. Ginsenoside Re, for example, regulates thermogenesis and hence improves vitality [68]. In heavy C57BL/6J ob/ob mice, a ginseng berry extract (150 mg/kg) was infused intraperitoneally for 12 days. The ob/ob mice treated with ginsenoside encountered a 15% decrease in food consumption and a 15% decrease in body weight (11.6 percent). Internal heat level (2.8%) and essentialness use (35%) both rose in development in ob/ob mice [68].

##### *Hoodia gordonii* (Hoodia)

*Hoodia gordonii* is a medicinal plant that has a significant ability to treat T2DM. P57AS3, an oxypregnane steroidal glycoside, is conveyed by *H. gordonii* and *H. pilifera*. P57AS3 was overseen into the third ventricle and diminished 24 h sustenance affirmations by 40–60% [69]. P57AS3, moreover, extended adenosine triphosphate (ATP) blend, suggesting that *H. gordonii* coordinates dietary affirmation and centrality balance. Food affirmation and weight were lessened at all estimations when glycosides 1 and 2, segregated from dried *H. Gordonii* stems, were offered orally to rodents in segments from 6.25 to 50 mg/kg for eight days, appearing differently from the control group [70]. Various elements influenced by *H. gordonii* fuse mitochondrial carnitine palmitoyltransferase-1 (CPT-1), thyroid synthetic substances, NPY, and affront-like progression factor-1 (IGF-1) [71]. Food confirmation and NPY levels were lessened restrictively when male Sprague Dawley rodents were given three unmistakable dosages (50, 100, and 150 mg/kg of body weight) of standard dissolvable concentrate from *H.gordonii* orally. Thyroid synthetic substances such as triiodothyronine (T3) and thyroxine (T4), similarly to CPT-1, were extended. CPT-1 is an oil destructive oxidation atom associated with the lipid processing framework, and its expanded levels infer better greasy destructive oxidation. As the carbohydrate processing framework advances, T3 and T4 stature mirror an expansion in energy utilization and glucose homeostasis. In this sense, *H. gordonii* might be useful in diminishing hunger, working on fast processing, and further developing starch absorption [72].

##### *Vaccinium* spp. (Blueberry)

Berries have long been known for their antioxidant effects. Blueberries have been demonstrated to have antioxidant, anti-obesity, and antidiabetic characteristics, as well as improving cardiovascular health [73,74]. Anthocyanins make up most of the mixtures in blueberries, with hydroxycinnamic destructive, flavonols, flavan-3-ols, folic destructive, nutrient C, and fiber making up the rest [75]. The concentrates from two cultivars of blueberries, “Centurion” and “Maru”, have been displayed to decrease food admission by instigating satiety [76]. Following six days of drinking blueberry water, rodents’ food admission and body weight definitively decreased. When C57BL/6 mice on the HFD drank water imbued with blueberry extract, contrasted with the HFD control group, body weight and fat were decreased [77]. Anthocyanins lowered the HFD group’s fasting blood glucose concentration to normal levels. Berry anthocyanins, rather than blueberries alone, are reported to have a considerably higher anti-obesity effect [77,78].

#### 5.1.2. Compounds Stimulate Energy Expenditure

Energy utilization includes thermogenesis, genuine development, and head centrality use [79]. The two sorts of fat tissue are white adipose tissue (WAT) and brown adipose tissue (BAT). WAT stores overabundant energy as oils, and BAT produces heat. Researchers are attempting to conclude how to utilize BAT to offer warmth to the body, with the ultimate objective of being utilized. Uncoupling proteins (UCP1, UCP2, and UCP3) are key cell stomach-related design controllers that keep responsive oxygen age [80]. UCP1 catalyzes flexible thermogenesis in mammalian BAT. UCP2 and UCP3 can be thermogenic when affected by the right effectors, and they regularly respond to adaptable thermogenesis [80].

##### *Nelumbo nucifera* (Indian Lotus)

*Nelumbo nucifera* has been used to cure a range of diseases for millennia. It has a wide range of medicinal qualities, including relief from fever, burning skin problems, and clutter removal [81]. The elimination of clutter has an effect on protein digestion, fat digestion, and thermogenesis in the stomach. When *N*. *nucifera* shoots were fed to HFD-fed mice for five weeks, alpha-amylase and lipase activity were lowered but fat digestion and UCP3 mRNA expression was increased in C2C12 myotubes [82]. The upregulation of UCP3, which is expressed in BAT and skeletal muscle, leads to enhanced thermogenesis. During growth, it removes *N. nucifera*, which contains eleven flavonoids, including the sesquiterpene eudesmane; thirteen megastigmanes; and eight alkaloids [83,84,85]. Flavonoids, for example, are hypolipidemic and restrain pancreatic lipase, glucosidase, and amylase. *N. ginseng* is a root vegetable, such as *P. ginseng*. *Nucifera* has been shown to promote vitality consumption [86].

##### *Capsicum annuum* (Chili Pepper)

The active element of *Capsicum annuum*, sometimes known as crimson chili peppers, is capsaicin. In Southeast Asia, China, and Latin America, it is commonly used as a flavoring [87]. Capsaicin has been demonstrated to promote thermogenesis by causing the adrenal medulla to release more catecholamines [88]. In vitro, capsaicin can extend the assertion of characteristics related to lipid catabolism and thermogenesis, such as smart synthetic lipase, CPT-1a, and UCP2 [89]. By uncoupling oxidative phosphorylation, UCP2 improves thermogenesis [88]. The mRNA level of UCP2 extends segments restrictively considering capsaicin [89]. Capsiate, which is available in CH-19 sweet pepper, has a structure that is similar to capsaicin yet comes up short on astringency [90]. Capsiate activates BAT, which enhances vitality consumption in humans [91]. According to in vivo studies [92], capsaicin enhances vitality usage in adults. Subjects were given either a regular meal with 2.56 mg of capsaicin or a placebo after being placed in a breathing chamber. A subsequent group was given similar conditions to the first, however with a 25% calorie limitation and either a similar amount of capsaicin or none by any means. Resting energy expenditure (REE) and diet-initiated thermogenesis (DIT) were higher in the calorie-confined capsaicin group than in the calorie-limited group. Fat oxidation was likewise raised in the calorie-limited capsaicin group. Capsaicin upgrades REE and DIT in a negative essentialness change, as indicated by this outcome. Capsaicin can be utilized to treat heftiness and weight decrease in those on a low-calorie diet.

#### 5.1.3. Compounds Regulate Lipid Metabolism

##### *Camellia sinensis* (Green Tea)

One of the main catechins in *C. sinensis* is epigallocatechin gallate (EGCG) [93]. At the point when HFD mice were given EGCG (3.2 g/kg thin down) for about four months, their bodyweight expanded, muscle versus fat rate diminished, and instinctive fat weight diminished in contrast with HFD control mice [94]. EGCG likewise decreased vascular opposition, plasma cholesterol, liver weight, and liver fatty substance levels (TG). Likewise, when contrasted with HFD control mice, transient EGCG treatment (3.2 g/kg eat less) for about a month diminished mesenteric fat weight and blood glucose. This finding proposes that EGCG is connected to a decrease in lipid total in the liver. In another investigation [95], EGCG decreased total cholesterol (TC) and low-density lipoprotein (LDL) cholesterol levels in HFD rodents with 1% EGCG supplementation, in contrast with HFD control rodents. The uptake of cholesterol in the digestive tract was additionally diminished in the EGCG group. When combined with caffeine in a meta-examination, a clinical report with EGCG and caffeine [96], EGCG decreased bodyweight and restricted bodyweight gained after weight reduction by expanding essentialness and fat oxidation. This discovery gave the idea that ECGC could help caffeine in expanding energy utilization.

##### *Vaccinium angustifolium* (Wild Blueberry)

Wild blueberries contain anthocyanin, an antioxidant polyphenol. When stout and skinny Zucker rats were fed a wild blueberry-enriched diet for eight weeks, the corpulent Zucker rats demonstrated an increase in dyslipidemia and changes in lipid digesting system features [97]. The stout group had considerably reduced levels of triacylglycerol (TAG) and triacylcarnitine (TC), but not the lean group. Moreover, in the stomach fat tissue of overweight mice, the articulation levels of PPAR and PPAR-interpretation parts of the lipid absorption framework were expanded in abdominal adipose tissue (AAT). After separation, SREBP-1 and FAS levels in the AAT and liver were lower. This information infers that wild blueberry advances lipid absorption through coordinating lipid processing-related attributes.

##### *Cinnamomum* spp. (Cinnamon)

Cinnamon is made from the *Cinnamomum* tree’s bark. Cinnamon extract enacts PPAR, which increments fat assimilation and opposition [98]. PPAR targets lipoprotein lipase (LPL), bunch parcel 36 (CD36), glucose transporter sort 4 (GLUT4), and acyl CoA oxidase. The expression levels of 3T3-L1 adipocytes increased during treatment with cinnamon extract. Right when C57BL/6J db/db mice were gavaged with cinnamon powder isolated in water, fasting hyperglycemia, free slick dangerous, LDL cholesterol, and aspartate aminotransferase (AST) levels all fell due to the execution of PPARs. In ob/ob mice, cinnamon further creates assault affectability, insulin-empowered locomotor activity, and glucose strength [99]. Water-dissolvable cinnamon concentrates might conceivably affect body synthesis and feature metabolic issues [100]. 

#### 5.1.4. Possible Therapeutic Compounds That Regulate Carbohydrate Metabolism

Although several studies suggest a link between carbohydrate digestion and weight gain, the glucose digestive system is often disregarded when it comes to weight increase. The Atkins tally calories group seemed to lose more weight for a half year in a clinical exploration looking at every day tally calories (a high-carb, low-fat, low-calorie) and Atkins thin down (a low-starch, high-protein, and high-fat, low-carb) groups [101]. Diminished sugar osmosis [102] and carb assimilation impediments [103] are generally productive ways to deal with further developed chubbiness through more prominent weight reduction and diminished weight acquisition.

##### *Camellia sinensis* (Teas)

By inhibiting alpha-amylase [104], alpha-glucosidase sodium-glucose transporters [105,106], and pancreatic lipase [107,108], black, green, and mulberry tea extracts may prevent carbohydrate absorption. In a human examination, black, green, and mulberry tea extracts were offered orally to strong people. Breath-hydrogen and 13CO_2_ levels were measured to decide starch retention. Volunteers were offered the decision of black (0.1 g), green (0.1 g), or mulberry (0.1 g) teas at each stage. Individuals ate lipid-and starch-rich dinners, just as lipid-just and carb-free dinners. Increased breath-hydrogen concentration was observed after suppers including fat, carbohydrate, and tea, indicating carbohydrate malabsorption. The carbohydrate-free dinners made with or without tea extract, on the other hand, showed no differences. This suggests that the compounds in tea extract may reduce carbohydrate retention [102].

##### *Glycine max* Merr (Soybean)

Soy has an assortment of effects against stoutness and is hostile to diabetic impacts [107]. Food upgraded with soybean isoflavones was used to help rodents and overweight SHR/N-cp rodents [108]. It was proposed that they ingest 20% casein, 20% casein with 0.1% soybean isoflavone mix, 20% casein with 0.1% probiotic blend, and 20% casein with 0.1% isoflavone and 0.1% probiotic blend. Isoflavones alone cut down plasma glucose, AST, and ALT in the two groups (alanine transaminase). AST and ALT are plasma particles identified within the liver. They are higher in hefty rodents than in lean rodents. The expanded starch content of the dinner raised AST and ALT levels, which expanded fat focus [109]. Soy isoflavones may perniciously affect carb processing, as indicated by these scientists.

### 5.2. Possible Therapeutic Compounds for Diabetes 

#### 5.2.1. Possible Therapeutic Compounds That Regulate Insulin Resistance

##### *Vaccinium* spp. (Blueberry)

Insulin resistance is improved by eating blueberries. Mice were fed freeze-dried whole blueberry powder supplemented with HFD for 8 weeks [110]. Blueberry-fed mice had lower blood glucose levels than the control group, according to the findings. The increase in adipose tissue macrophage (ATM) appears to have a role in the development of insulin resistance [111]. The M1 macrophage causes tissue injury and inflammation [112]. Cell growth and tissue healing are triggered by M2 macrophages. M1 and M2 ATM numbers were lower in mice fed an HFD-containing blueberry compared to those fed HFD alone. Furthermore, mice on the blueberry-rich HFD had decreased levels of ATM-related inflammatory gene expression in epididymal adipose tissue, including TNF-α, and monocyte chemotactic protein-1 (MCP-1). Another study found that combining blueberry and Labrasol in the mouth can lower blood glucose levels [74]. Blueberry was made in one study by fermenting with *S. vaccinia* [73]. KKAy mice were given the fermented blueberry both acutely and chronically. When compared to the control group, both the acute and chronic administration groups demonstrated a drop in blood glucose levels. PPAR has a variety of activities, including glucose and lipid metabolism [113]. The PPAR-alpha and PPAR activity of Zucker rats given blueberries were increased [113]. Furthermore, PPAR agonists promote fat metabolism and reduce insulin resistance [113]. This could indicate that blueberry extract reduces insulin resistance by activating the PPAR receptor [113]. Another study found that obese or T2DM patients who consumed 22.5 g blueberry twice a day for 6 weeks had lower insulin resistance than those who took a placebo [114].

##### *Glycyrrhiza glabra* (Liquorice)

*Glycyrrhiza glabra* extracts include amorfrutins, which can be used to treat diabetes. HFD-induced obese mice were given chemically produced amorfrutins [115]. When amorfrutins were given, blood glucose, plasma insulin, and body weight were all lowered. The plasma insulin levels of mice on a low-fat diet (LFD) were also compared. HFD-fed mice had greater plasma insulin levels than LFD-fed mice. Mice fed an HFD with amorfrutins, on the other hand, had plasma insulin levels comparable to mice fed an LFD [115].

KKAy mice were given an ethanol extract of *Glycyrrhiza* orally [116]. Blood glucose levels, body weight, and abdominal fat tissue were all lowered. *Glycyrrhiza* extract binds to PPAR according to the results of a binding affinity assay [115,116]. PPAR is implicated in the control of insulin sensitivity and glucose homeostasis, among other things [117,118]. This implies that *Glycyrrhiza* extract can help with insulin sensitivity and hyperglycemia [31].

##### *Trigonella foenum-graecum* (Fenugreek)

Fenugreek seeds (*Trigonella foenumgraecum*) are used as a food supplement and have a long history of therapeutic use for inducing labor, aiding digestion, and overall health [119]. Fenugreek seed extract has been found in animal experiments to reduce blood glucose levels [120]. It is thought to be a viable treatment for diabetes and its side effects [119].

Insulin resistance is reduced as a result of this plant’s glucose-lowering activity [121]. The active antidiabetic chemicals in fenugreek have been discovered as diosgenin, GII, galactomannan, trigoneosides, and 4-hydroxyisoleucine. However, nothing is known about these chemicals’ processes [122]. Diosgenin, for example, has been demonstrated to inhibit adipocyte differentiation and inflammation, hinting that it can help with insulin resistance [123]. According to a clinical investigation, fenugreek improves insulin sensitivity and thus hypoglycemia management [124].

In India, fenugreek seeds are used as a spice and to make bread with wheat and maize flour. It has also been used for medicinal purposes for a long time. When diabetic patients were given fenugreek seeds, blood glucose levels were lowered and plasma insulin levels were increased [125]. Patients with non-insulin-dependent diabetes were studied in another study [126]. For ten days, research participants were fed bread with similar amounts of ground fenugreek seed. The fenugreek group had lower fasting blood glucose and serum insulin levels than the control group. Another 20-day clinical trial was conducted. The fenugreek group also had lower fasting blood glucose levels [127]. This finding shows that fenugreek could be utilized to treat diabetes [127].

##### *Cinnamomum* spp. (Cinnamon)

Cinnamon has been used in traditional medicine to cure rheumatism, wounds, diarrhea, headaches, and colds, in addition to being a herbal food preservative and spice [128]. Cinnamon supplements are also used to treat asthma, arthritis, cancer, high cholesterol, T2DM, and other metabolic diseases. Cinnamon has been linked to diabetes treatment in a number of studies. Cinnamon may have similar actions to insulin [129].

Cinnamon appears to modulate insulin signaling in rat adipocytes in an in vitro study [130], proving that cinnamon enhances the impact of insulin. As the concentration of wortmannin increased, glucose oxidation by insulin and cinnamon decreased. Wortmannin is a fungus that suppresses the action of phosphoinositide 3-kinase (PI3K). Cinnamon, through activating PI3K in insulin signaling, has characteristics comparable to insulin. Cinnamon activates PI3K, which causes insulin signaling. Cinnamon also activates tyrosine phosphatase in insulin signaling, according to a protein tyrosine phosphatase (PTP-1) experiment. Cinnamon regulates the insulin receptor, GLUT4, and tristetraprolin (TTP) expression in 3T3-L1 adipocytes, according to research [131]. Two types of cinnamon were employed in this study: aqueous extract and cinnamon polyphenol. The water extract did not stimulate insulin receptor protein expression, but the cinnamon polyphenol did. GLUT4 protein expression increased when both water extract and cinnamon polyphenol were administered in the therapy [132]. TTP is an inflammatory protein whose mRNA expression was shown to be elevated in a mouse fibroblast cell line after insulin therapy. TTP protein and mRNA expression levels were both raised when both water extract and polyphenol were applied [130]. Cinnamon can increase insulin receptor and GLUT4 protein expression, according to the findings. This suggests that cinnamon may enhance insulin activity and may potentially have a similar effect to insulin. As a result, cinnamon may be able to help with insulin resistance, which is one of the numerous signs of obesity and T2DM.

##### *Gymnema* *sylvestre*

It has long been recognized that the Indian medicinal plant *Gymnema sylvestre* possesses antidiabetic properties. This plant’s extracts have been shown to exert a variety of antidiabetic effects, including a reduction in insulin requirements, improved blood glucose homeostasis, better control of hyperlipidemia, and an increase in serum C-peptide levels, all of which have been linked to the plant’s antidiabetic properties [133,134]. B cells in pancreatic islets from STZ-inducing diabetic rats can be revived by the use of water-soluble alcohol extracts from *G. sylvestre* [133]. Additionally, it has been shown that water-soluble alcoholic extracts of *G. sylvestre* leaves enhance insulin release from pancreatic B cells in various animal models of hyperglycemia and diabetes. When tested on alloxan-induced diabetic rats, the dried powder of *G. sylvestre* was found to regulate blood sugar homeostasis and to boost the activity of insulin-dependent pathway glucose utilization enzymes [135]. Siddiqui et al. recently examined the chemistry and pharmacology of *G. sylvestre* [134]. In spite of the fact that crude combinations of *G. sylvestre* have been isolated and evaluated for hypoglycemic action and extracts from leaves have been found to be efficient in regulating the absorption of sugar, the particular components responsible for activity have not been established [134]. To better understand how *G. sylvestre* leaf extracts can help diabetics with type 2 hyperglycemia, researchers Baskaran et al. [136] conducted a study. The extract reduced blood glucose, glycosylated hemoglobin, and glycosylated plasma proteins with a decrease in the dosage of conventional drugs, according to researchers’ findings. Some individuals were able to stop taking conventional medications and even keep their blood glucose levels stable by taking extracts only. A water-soluble extract of the leaves of *G. sylvestre* lowered insulin requirements, improved blood glucose homeostasis, better controlled hyperlipidemia, reduced serum amylase activity, and boosted b-cell activities in insulin-dependent patients after extended dosing [133].

Human clinical investigations on the effects of Gymnema on T1DM and T2DM are few, and most of them have flaws in their methodology. The fact that many Gymnema trials use patented multiple herb combinations further limits the number of studies that are eligible. Gymnema single herb trials are described in the following five papers.

A case series of eight T2DM patients given 10 mg of dried *Gymnema sylvestre* leaf powder for 21 days found a statistically significant reduction in fasting blood glucose of 50.5 mg/dL and a reduction in two-hour post-prandial blood glucose levels of 40.5 mg/dL at 21 days (*p* < 0.05), as compared with the same patients prior to treatment with Gymnema. During the same 21-day period, the patients’ body weight increased by 0.9 kg, which was deemed to be non-significant. There was no control group in this study [137].

In 1992, the same author presented a randomized controlled experiment with three research aims: For a total of 21 days, 16 healthy volunteers and 43 T2DM patients were given Gymnema or tolbutamide (an oral hypoglycemic medication at a normal therapeutic dose). The patients were not randomly assigned to groups, and neither the investigators nor the patients were blinded. The non-diabetic patients experienced a significant reduction in fasting blood sugar (FBS, from 80.8 mg/dL to 71.6 mg/dL) on day 7. The diabetic patients in the Gymnema-treated group experienced significant reductions in both FBS (152 mg/dL to 133 mg/dL) and post-prandial blood sugar (PPBS, 215 mg/dL to 142 mg/dL) at 21 days. On day 7, the diabetic patients in the tolbutamide therapy group had significantly lower FBS and PPBS, but not on day 14. Decreases also were reported in total cholesterol for both diabetic and non-diabetic patients (284 mg/dL to 244 mg/dL and 217 mg/dL to 200 mg/dL, respectively). P-values were not provided for this short, non-randomized study [138].

For 20 months, 47 T2DM patients were followed in a lengthier controlled clinical trial. Each patient was already taking oral hypoglycemics and was maintaining a consistent treatment regimen with only partial control. The normal treatment was continued for half of the patients. For the other half, 400 mg of GS4, a Gymnema extract, was added to their daily routine. The average HBA1C in the GS4 group fell from 12% to 8.5%; FBS decreased as well, from 174 mg/dL to 124 mg/dL; total cholesterol and triglycerides improved from 260 mg/dL to 231 mg/dL and from 170 mg/dL to 140 mg/dL, respectively. All results were significant at the *p* < 0.001 level. Except for the Gymnema, 23 percent of the patients in the GS4 therapy group were able to cease taking all hypoglycemic medicines. There are a few caveats to be aware of when interpreting this study. The patients were not assigned to treatment groups at random, and there were disparities in fasting glucose levels at the start. They were, however, fairly well matched in terms of weight and BMI [136].

Ten healthy volunteers and six T2DM patients were given 2 g of an aqueous decoction of the shade-dried leaves of Gymnema three times per day in a modest, short-term trial. Significant reductions in both FBS and PPBS were demonstrated. After 10 days, significant reductions were seen in the FBS of both normal (80.2 mg/dL to 69.2 mg/dL; *p* < 0.05) and T2DM (T2DM135.7 mg/dL to 110.7 mg/dL, *p* < 0.02) patients. Significant reductions were also demonstrated in 30 min PPBS (220 mg/dL to 180.7 mg/dL, *p* < 0.05) and two-hour PPBS (152.7 mg/dL to 121.1 mg/dL, *p* < 0.01) in the Type 2 group [139].

Only one study evaluated the effectiveness of Gymnema in T1DM (*n* = 63, 8–30 years old). Thirty-seven patients received their usual and customary care, whereas 27 insulin-dependent patients received 400 mg of Gymnema daily for eight months in addition to their usual insulin regimen. In the supplemented group, the average insulin need dropped from 60 uNPH/d to 45 uNPH/d. At least one episode of hypoglycemia occurred in all Gymnema patients, prompting insulin dosage reductions. Unfortunately, 40% of the patients withdrew before the completion of the trial due to one patient’s severe hypoglycemia and practical difficulties with follow-up for the rest [133].

These studies, taken together, strongly demonstrate that Gymnema has a favorable effect on blood sugar in both T1DM and T2DM. This, together with the beneficial effect on blood lipids, is especially reassuring given the high likelihood of dyslipidemia in diabetic individuals. However, as is common in preliminary data, these trials include design and analysis flaws. To confirm the effects found in the Indian trials, larger, well-designed clinical trials should be conducted.

#### 5.2.2. Possible Therapeutic Compounds Regulate β-Cell Function

##### *Ervatamia microphylla* (Kerr)

Conophylline is a substance derived from the *Ervatamia microphylla* plant. It contains antidiabetic properties. When streptozotocin (STZ)-induced diabetic rats were given conophylline orally, their blood glucose levels were lowered [140]. Pancreatic β-cell proliferation was stimulated by conophylline [141]. According to a study on pancreatic stellate cells, conophylline reduces stellate cell activation [142]. Furthermore, conophylline lowered blood glucose levels while increasing plasma insulin levels in Goto-Kakizaki rats in an in vivo trial [142].

##### *Anoectochilus roxburghii* (Jewel Orchid) 

Kinsenoside is a chemical found in the jewel orchid *Anoectochilus roxburghii.* In STZ-induced hyperglycemic rats, the oral treatment of kinsenoside lowered blood glucose levels [143]. Plasma insulin levels were higher in the kinsenoside group than in the control group due to larger pancreatic β-cells [31].

##### *Nymphaea* *stellata*

Initially, Nymphayol was used to isolate from *Nymphaea stellata*. This chemical was found to induce the partial production of pancreatic islet cells in one study [144]. In diabetic rats, the oral treatment of Nymphayol dramatically reduced blood glucose levels and boosted insulin content. Nymphayol also greatly boosted the number of cells [144].

#### 5.2.3. Compounds with Multiple Antidiabetic Activities

##### *Capsicum frutescens* (Solanaceae)

After 4 weeks of treatment, *Capsicum frutescens* enhanced serum insulin concentration in streptozotocin-induced T2DM rats fed a high-fat (HF) diet. In the experimental procedures, the data from this study imply that 2% dietary *Capsicum frutescens* is insulinotropic rather than hypoglycemic [22,87].

##### *Momordica charantia* (Cucurbitaceae)

Diabetic rats treated with *Momordica charantia* fruit juice showed a significant drop in blood glucose levels and an increase in plasma insulin concentration. The result was attributed to a higher number of beta cells in treated mice compared to untreated animals. Momordicin, charantin, and a few other substances isolated from other portions of this plant, such as galactose-binding lectin and insulin-like protein, have been demonstrated to have insulin-mimetic activity [145,146].

The aqueous extract of unripe *M. charantia* fruits has also been demonstrated to partially induce insulin release from the isolated beta-cells of obese-hyperglycemic mice, implying that the insulin-releasing effect is caused by membrane processes being disrupted [147]. *M. charantia* enhances pancreatic insulin secretion by increasing the renewal of partial cells in the pancreas or allowing the recovery of partially damaged cells [22].

A human clinical investigation was undertaken with 18 mature-onset diabetes patients [148]. Thirty minutes before blood glucose levels were measured, the patients were provided *M. charantia* juice or a vehicle. Patients who took *M. charantia* juice had lower blood glucose levels than the vehicle control group. These findings suggest that *M. charantia* can lower blood glucose levels in diabetic mice and patients [31].

##### *Vitis vinifera* (Grape Vine) 

Resveratrol is a polyphenol that is found in the extract of *Vitis vinifera* [149]. It possesses a wide range of bioactivities, including hepatoprotective, anti-cancer, anti-inflammatory, immunomodulatory, antidiabetic, and other properties [132,150,151]. In many rodent models, resveratrol has been shown to treat diabetes [152,153] and its complications [154,155,156,157,158,159]. Evidence suggests that resveratrol has numerous modes of action when used as a T2DM treatment. In db/db mice, this chemical can activate AMPK and downstream pathways, resulting in a reduction in insulin resistance [160,161]. It also reduced IAP-induced cell death in pancreatic cells in culture [162] and STZ-treated animals [163].

Furthermore, resveratrol increased glucose-mediated insulin production in cells by activating SIRT1 [164], one of resveratrol’s biological targets [165,166]. Resveratrol has been shown to improve glycemic control in T2DM patients in a clinical investigation [149].

In a HepG2 cell line, *V. vinifera* extract decreases glycogen phosphorylase b activity [167]. Glycogen phosphorylase b is an enzyme that transforms glycogen to glucose-1-phosphate and is involved in the rate-limiting stage of glycogenolysis. Because T2DM patients have higher hepatic glucose levels, glycogen phosphorylase is a good target for treatment [117]. So, inhibiting glycogen phosphorylase activity can lower hepatic glucose levels in T2DM patients. Overall, *V. vinifera* extract can help T2DM patients with high blood and liver glucose levels [31].

### 5.3. Possible Therapeutic Compounds for Both Obesity and Diabetes

Obesity and diabetes share certain basic characteristics and are intimately linked. In the 1970s, the term “diabesity” was coined to stress the close link between obesity and diabetes. Some diabetes medications, on the other hand, may contribute to obesity. Sulfonylureas, for example, can cause weight gain as a side effect [168]. Obesity and diabetes both have no viable therapies that are free of negative effects. Because phytogenic substances have fewer negative effects than chemical medications, we rely on them to treat obesity and diabetes [10].

In female mice, genistein reduces food intake, body weight, and fat pad weight while increasing the apoptosis of adipose tissue in several animal investigations [169,170]. A large number of studies on preadipocytes and adipocytes have been performed to confirm these findings and to further understand the mechanisms engaged at the molecular level. Reduced food intake and body weight loss mediated by genistein appear to be at least partially owing to decreased leptin production, the most significant adipose-derived hormone that regulates energy intake and expenditure via appetite and metabolism regulation [171].

Genistein improved glucose and lipid metabolisms, raised insulin levels, and maintained pancreatic b cells in studies on C57BL/KsJ-db/db mice [172] and non-obese diabetic mice [173]. Glycerol-6-phosphate dehydrogenase was activated, while enzymes involved in hepatic gluconeogenic and lipogenic activities, such as glucose-6-phosphatase, phosphoenolpyruvate carboxykinase, fatty acid b-oxidation, and carnitine palmitoyltransferase, were downregulated. Furthermore, genistein potentiated glucose-stimulated insulin secretion in both insulin-secreting cell lines and mouse pancreatic islets [174], as well as inducing the phosphorylation of Erk1/2 and protein expression of cyclin D1, a major cell-cycle regulator essential for b-cell growth in human islets [174]. These effects were independent of genistein estrogenic-like activity and were not mediated through the protein tyrosine kinase (PTK) or nitric oxide signaling pathways [175]. Furthermore, genistein had no effect on ATP-sensitive potassium channel activity.

Insulin resistance is improved [100,110], the appetite is suppressed [76], and lipid metabolism is regulated by *Vaccicum* spp. [97]. Blueberry anthocyanins could be useful in the treatment of obesity and diabetes. Food intake, body weight gain, body fat, and blood glucose levels were all reduced by blueberry water extract, which also activated PPARs [77]. Furthermore, anthocyanins found in *V. angustifolium* (wild blueberry) ameliorate dyslipidemia via modulating the genes involved in lipid metabolism [97].

Obesity and diabetes are affected by *Glycyrrhiza* extract. Hyperglycemia can be improved with this phytogenic substance [176]. The extract of *Glycyrrhiza glabra* boosted oxidation while decreasing acetyl-CoA production [168]. *Glycyrrhiza* extract may also help to reduce body fat, according to clinical investigations [168,177,178,179].

Capsicum activates BAT, which causes thermogenesis [91,180]. While capsaicin’s effect on energy expenditure is unknown, it appears to treat hyperglycemia by boosting plasma insulin levels [181].

Hyperglycemia [182] and hyperlipidemia [183] can both be improved with *M. charantia* extract. Adipocyte hypertrophy is suppressed by *M. charantia* extract, and lipogenic gene expression, such as FAS, ACC-1, and LPL, is reduced [183]. As a result, *M. charantia* may act as both a lipogenesis inhibitor and a lipolysis stimulator. Hyperlipidemia and hyperglycemia may be reduced by *M. charantia* extract [31].

*Cinnamomum* extract can help to reduce blood sugar levels [99,131]. Cinnamon extract is thought to have an insulin-mimetic action or to increase insulin activity. *Cinnamomum* extract appears to have a fat-burning effect [98,100]. As a result, cinnamon extract has the potential to be used to treat obesity as well as diabetes [31].

## 6. Different Therapeutic Targets of Diabetes, Treating with Herbal Products

**Figure 4:**
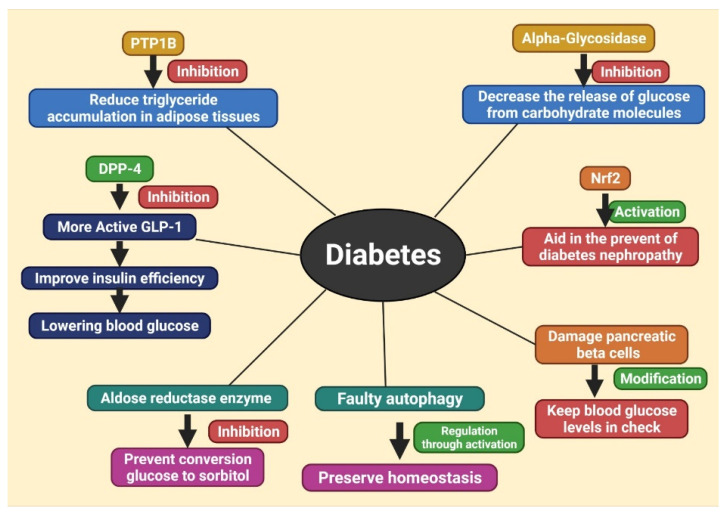
Different ways of treating diabetes.

Herbal products are the most abundant source of leads for developing new pharmacological entities, with a wide range of therapeutic indications and chemical structures [184]. Blood glucose is high in diabetes patients because their bodies cannot generate enough insulin or react effectively to this hormone [185]. Several therapeutic targets against diabetics with disease-causing effects have been discovered. Different ways of treating diabetes are depicted in Figure 4. Compared to synthetic antidiabetic medicines, natural compound-derived therapies are more readily accessible, expensive, and have fewer adverse effects [186]. Table 1 represents the herbal therapeutics with their mode of action against diabetes.

### 6.1. Inhibition of DPP-4

The DPP-4 enzyme, also known as adenosine deaminase binding protein or CD26, inactivates oligopeptides such as glucagon-like peptide-1 (GLP-1) by eliminating N-terminal dipeptides. It is distributed throughout the body and is prevalent in endothelial cells [187,188,189]. The body has two isoforms of DPP-4: the membrane DPP-4 (mDPP-4), which consists of a full-length DPP-4 peptide, and the soluble DPP-4 isoform (sDPP-4). GLP-1 controls the sensitivity and secretion of insulin and is generated by gut cells. With a half-life of under 2 min, it is rapidly metabolized into the inactive GLP-1 amide. By reducing sDPP-4 activity, more active GLP-1 can be maintained, improving insulin efficiency and therefore lowering blood glucose. The most prevalent types of DPP-4 inhibitors include naturally occurring flavonoids, phenolics, peptides, and terpenoids [187].

### 6.2. Inhibition of Protein Tyrosine Phosphatase 1B (PTP1B)

The protein tyrosine phosphatase 1B (PTP1B) enzyme regulates tyrosine phosphorylation in cells and has an essential function in negatively regulating insulin signal transmission. Its inhibition substantially reduces triglyceride accumulation in adipose tissues in the context of excess nutrition. PTP1B is thus a possible target for T2DM [190,191]. The allostery of PTP1B is controlled via dynamic and confirmative alterations, whereas its principal catalytic activity is driven by stiff conformational changes. As a consequence, it may block via the active site of allosteric inhibitors [192].

### 6.3. Inhibition of α-Glycosidase

The α-glucosidase enzymes hydrolyze starch to simple sugars that help the body to digest dietary carbohydrates and starch and thus increase the levels of blood glucose for intestinal absorption [193]. Inhibiting this metabolic enzyme decreases the release of glucose from carbohydrate molecules [194]. To the active site of α-glucosidase, these enzyme inhibitors create a more stable complex, thereby decreasing the hydrolysis of carbohydrates and maintaining hyperglycemic conditions [195]. Several chemical compounds from mushrooms or antioxidant compounds such as tannic acid from different herbal products play inhibitory roles against α-glucosidase enzymes [194,196].

### 6.4. Activation of Nrf2

Nuclear factor erythroid 2 (Nrf2) is a protein that combats oxidative stress and comprises seven functional domains. These are ARE and sMAF (Neh1), Keap1 (Neh2), CHD6-binding (Neh3), trans-activating, and CBP-binding areas (Neh4 and Neh5), ß-transducin repeat binding protein (ß-TrcP) (Neh6) and RXRα domain binding (Neh7). Under stress, Nrf2 leaks out of the proteasomal degradation machine to accumulate and move from the cytosol to the nucleus. Insulin resistance is caused by oxidative stress, and nitrosative stress and elevated phosphorylation levels of extracellular signal-related kinase may suppress cardiac Nrf2 activity. The activation of Nrf2 may aid in the prevention of diabetic nephropathy. Nrf2 departs from Keap1 and enters the nucleus, where it binds to genes that code for antioxidant enzymes [197,198,199]. The Nrf2 protein may be activated by various extracts and phytochemical substances found in herbs and vegetables.

### 6.5. Modification of Pancreatic Beta Cells

Insulin is an anti-hyperglycemic hormone produced by beta cells in the pancreas that helps to keep blood glucose levels in check [197]. As a consequence of the destruction to the pancreatic beta cells, insulin shortage occurs, leading to hyperglycemia, which could be either T1DM or T2DM. The activation of T cells or Immunological agents, such as cytokines and macrophages, may damage pancreatic beta cells in T1DM. In T2DM, however, excessive glucose, cholesterol, or inflammatory mediators may harm pancreatic beta cells. Several herbal products have been shown to improve pancreatic beta-cell regeneration and insulin production and inhibit pancreatic beta-cell apoptosis [198]. 

### 6.6. Inhibition of Aldose Reductase Enzyme

In hyperglycemia, aldose reductase (AR) is an NADPH-dependent Oxidoreductase and a major enzyme in the polyol pathway, converting glucose to sorbitol. Sorbitol cannot diffuse readily through the cell membrane, resulting in diabetic problems and a variety of cellular functional impairments. However, under normal physiological conditions, the hexokinase enzyme converts glucose to glucose-6-phosphate [200,201,202]. Flavonoids from herbal food sources especially have the inhibitory activity of the aldose reductase enzyme [203]. 

### 6.7. Regulation of Autophagy

**Figure 5:**
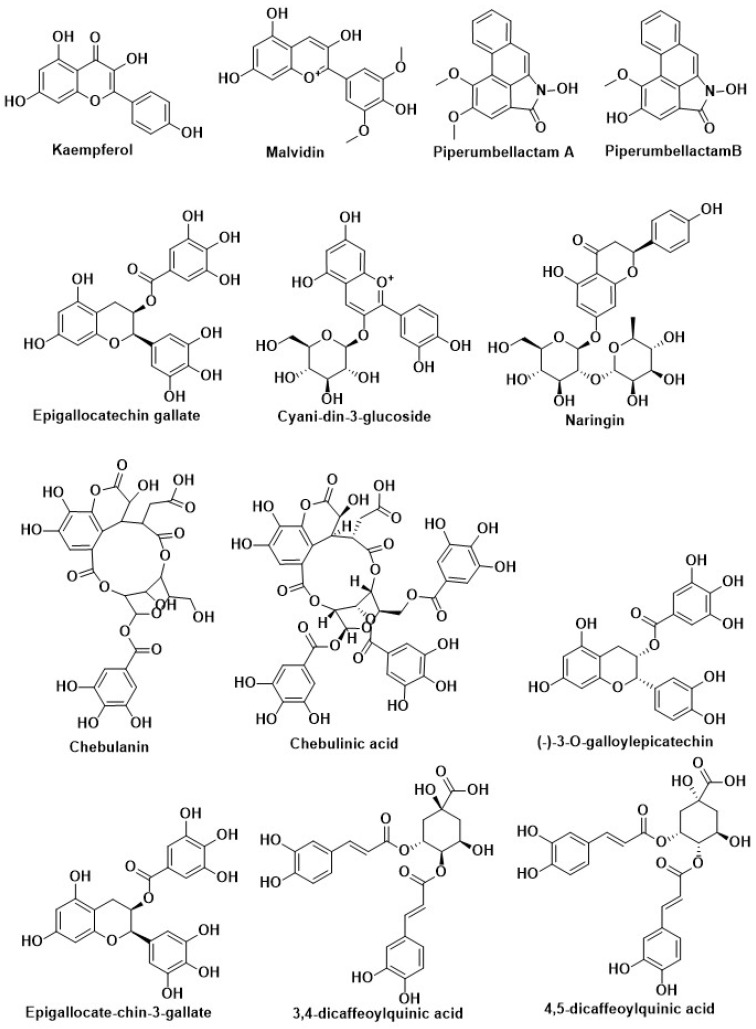

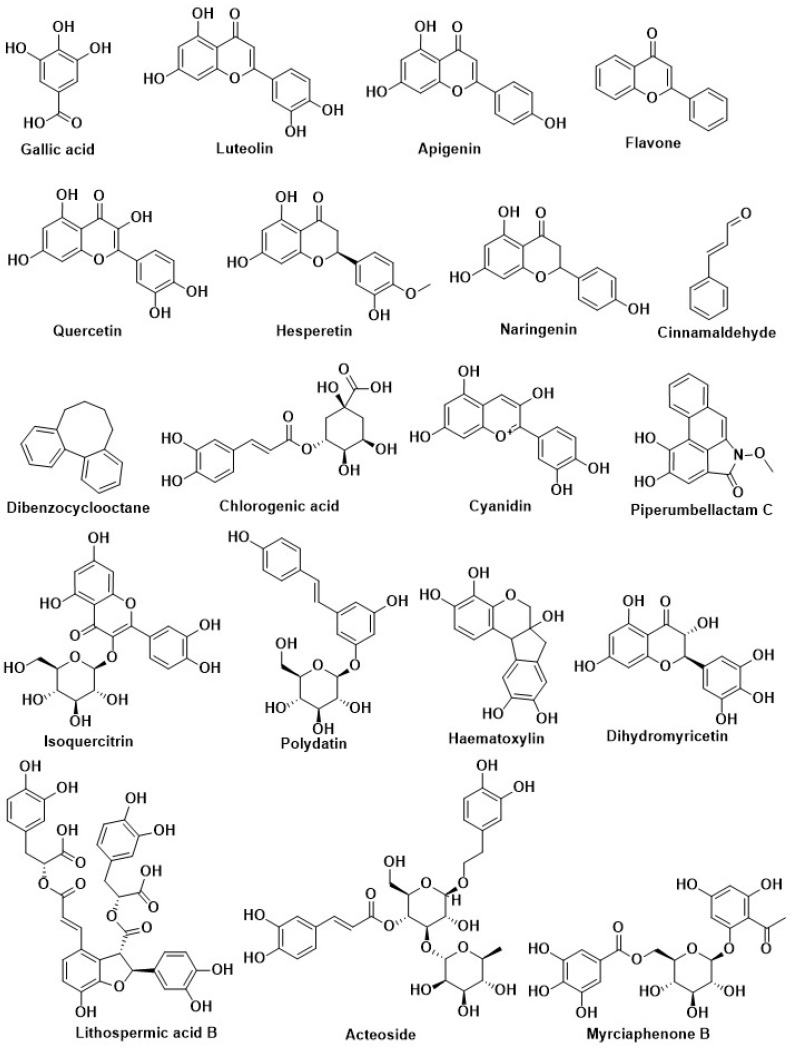

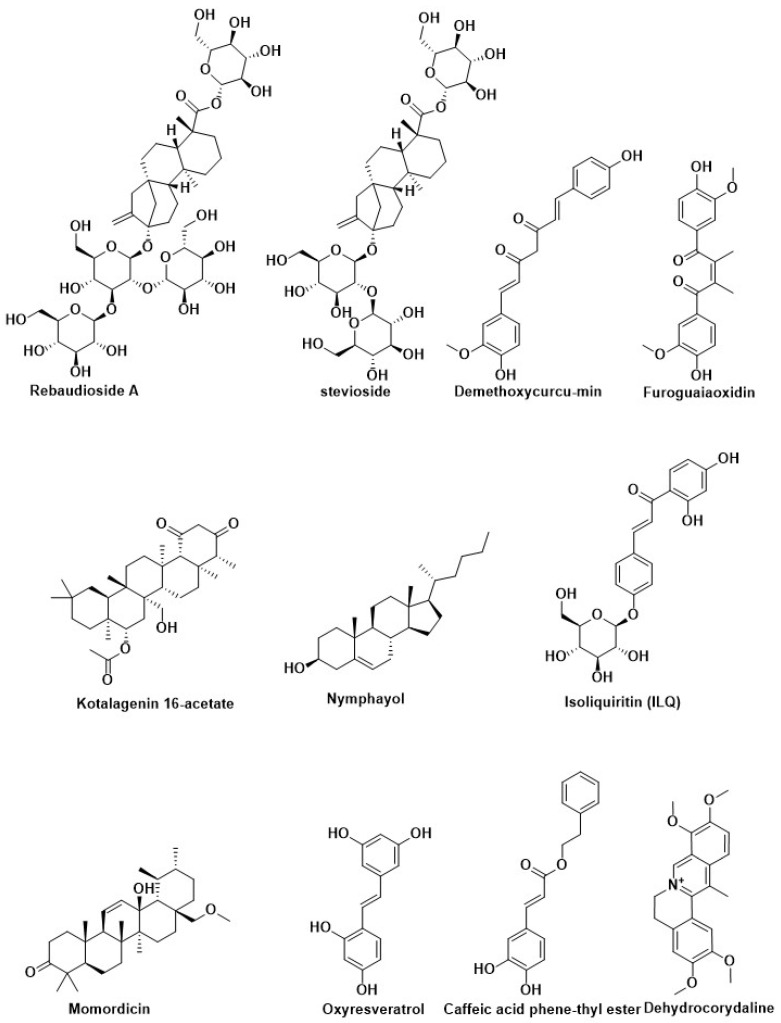

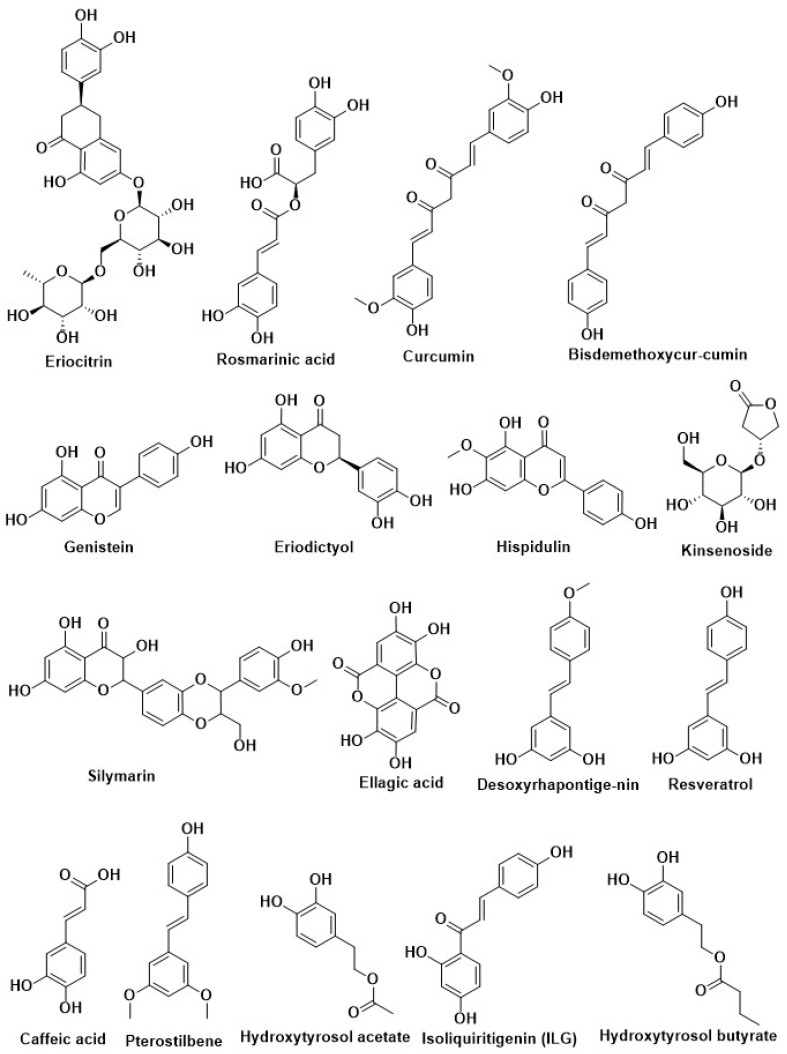

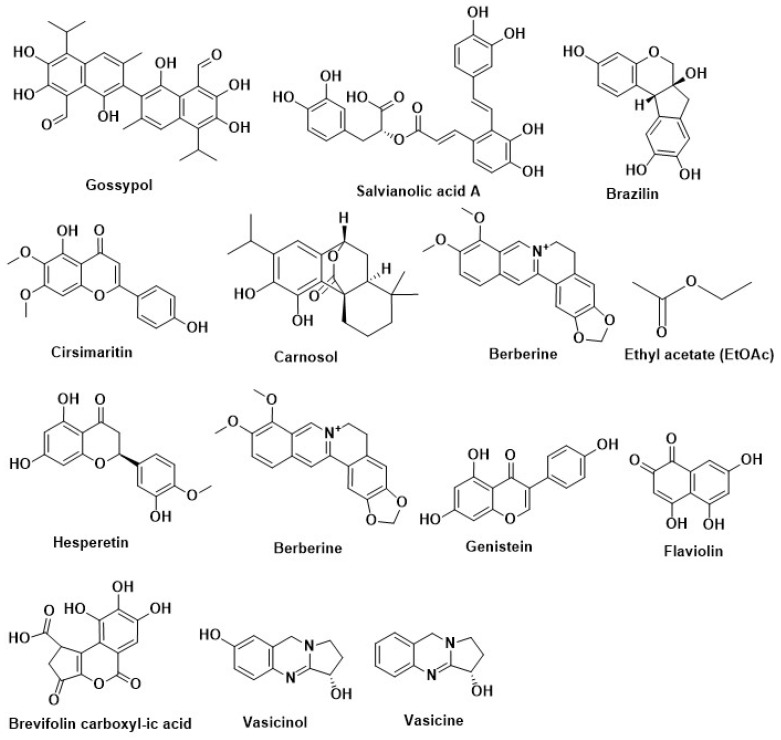
Chemical structures of phytocompounds against diabetes.

Autophagy is a lysosome-dependent homeostatic mechanism that contributes to the preservation of homeostasis in the cells and tissues. As a consequence, faulty autophagy contributes considerably to the development of metabolic disorders of carbohydrates, lipids, and proteins, such as T1DM and T2DM. People with diabetes who do not have enough insulin or cannot respond to it will have hyperglycemia and impaired autophagy. Therefore, autophagy offers a novel therapeutic target for diabetes, as activating autophagy has a range of benefits. AMPK and mTOR work together to act as essential regulators that monitor and control the amount of cell energy. It is generally assumed that inhibiting mTOR or activating AMPK is an efficient way to induce autophagy. The main anticipated herbal products that can trigger autophagy include resveratrol, berberine, quercetin, dihydromyricetin, and epigallocatechingallate [185] (Table 1 and Figure 5).

## 7. Different Therapeutic Targets of Obesity, Treating with Herbal Products

**Figure 6:**
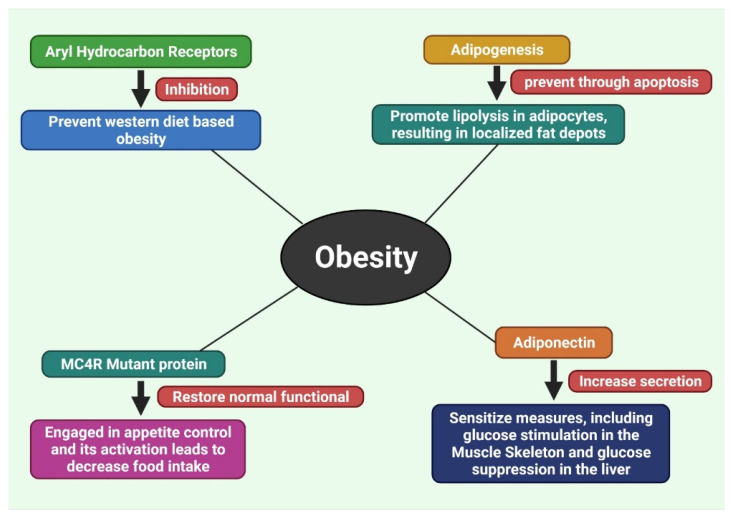
Different ways of treating obesity.

**Figure 7:**
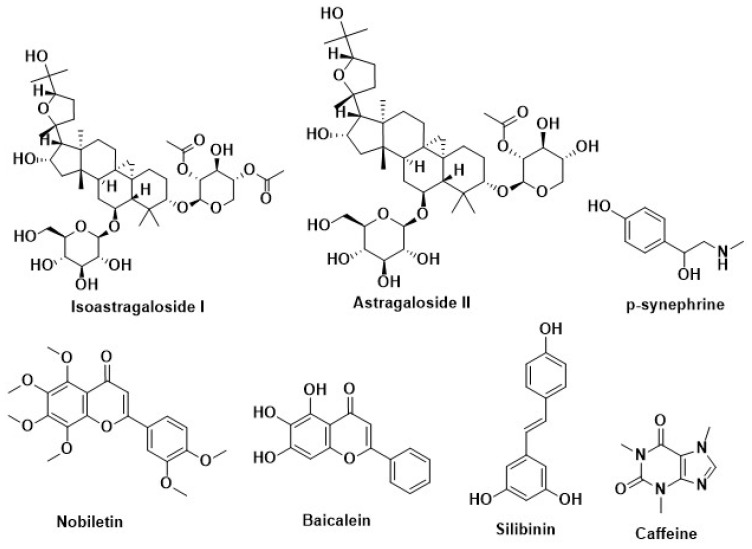
Chemical structures of phytocompounds against diabetes.

Obesity is a complicated and multifaceted illness, and it is prevalent in the world’s population, which is shifting away from the prior prevalence of infectious diseases and malnutrition. Obesity is a consequence of genetic predisposition, as well as a large number of high-energy meals being available, and a reduction in the physical activity required due to technological advancements. The idea that obesity is just a cosmetic issue for certain people is no longer valid since it is a worldwide pandemic [33,210]. The drugs and medicines from traditional Western medicine have some effectiveness in treating this illness. Certain chemicals such as phenolics, flavonoids, terpenoids, and other secondary metabolites are known to be present in plants and are known to have the ability to prevent obesity. These secondary metabolites are nanoparticle-encapsulated to improve their efficacy against obesity by increasing their target selectivity and effectiveness. This treatment targets secondary metabolites that are encased in nano-scaffolding and are not yet developed. They seem to target obesity by reducing lipid- and carbohydrate-metabolizing enzymes, blocking adipogenesis, and improving energy metabolism [33]. Different plant extracts with different anti-obesity effects are presented in Table 2 and the different targets for treating obesity are depicted in Figure 6 and Figure 7.

### 7.1. Inhibition of Aryl Hydrocarbon Receptors

The aryl hydrocarbon receptor (AhR) is an evolutionarily fundamental protein that functions as a ligand-activated transcription factor that detects the presence of external chemicals such as Persistent organic pollutants (POPs) and activates the cytochrome P450 enzymes necessary for their elimination from the body [211,212]. AhR may have an indirect effect on adipogenesis by modulating PPARγ expression and the production of some POPs with obesogenic activity. The activation of AhR impairs glucose metabolism, glucose tolerance, and insulin levels, thus increasing the risk of developing diabetes mellitus. Adipose tissue-specific AhR activation increases inflammation and impairs glucose and insulin tolerance [212]. Lipids and lipid derivatives, such as oxidized low-density lipoproteins (OxLDL), have been discovered as AhR agonists, which means that the saturated fatty acids found in a typical Western diet activate AhR and contribute to obesity and inflammation in C57B1/6 J mice [213,214]. Mice with low-affinity AhR alone are less likely to be obese, with a different fat mass, liver physiology, and hepatic gene expression than high-affinity AhR mice [215]. Environmental pollutants such as dioxins enter the body mostly through food and produce a variety of harmful effects via AhR transformation [216]. The inhibition of the AhR prevents Western diet-based obesity [214,217]. Lutein (IC_50_ = 3.2 µM), chlorophyll a (IC_50_ = 5.0 µM), chlorophyll b (IC_50_ = 5.9 µM), and (-)-Epigallocatechin gallate (IC_50_ = 1.7 µM) from green tea leaves protect against dioxin toxicity through the suppression of AhR transformation [85,216].

### 7.2. Inhibition of Adipogenesis by Methylxanthine

Adipocytes grow from fibroblast-like preadipocytes during adipogenesis. Many transcription factors, including the CCAAT/enhancer-binding protein (C/EBP) gene family and peroxisome proliferator-activated receptor (PPAR), must be activated sequentially during adipogenesis. These cells must go through two stages to mature: adipocyte determination and adipocyte differentiation. Several variables have been found. Some stimulators include PPAR γ, insulin-like growth factor I (IGF-1), macrophage colony-stimulating factor, fatty acids, prostaglandins, and glucocorticoids. Glycoproteins, transforming growth factor-β (TGF-β), inflammatory cytokines, and growth hormones are all inhibitors. Aside from these, age, gender, and lifestyle may all influence the process in some manner. Obesity is caused by an increase in the number and size of adipocytes. Adipogenesis may cause central obesity (abdominal fat depot) or peripheral obesity (subcutaneous tissue) [218]. Herbal products can cause apoptosis, prevent adipogenesis, and promote lipolysis in adipocytes, resulting in localized fat depots and peripheral obesity if it happens in subcutaneous tissue [219]. Several herbal products, such as *Silybum marianum*, *Citrus aurantium*, *Taraxacum officinale*, resveratrol, *Curcuma longa*, caffeine, etc. reduce differentiation and increase lipolysis and apoptosis [220].

### 7.3. Recover the Disruption of Melanocortin 4 Receptor *(MC4R)* Protein

The melanocortin 4 receptor (MC4R) is a seven-transmembrane G-protein-coupled receptor (GPCR) that is encoded by a single exon gene on chromosome 18q22. MC4R is expressed in the hypothalamic paraventricular nucleus, a region of the brain that is intricately engaged in appetite control, and its activation leads to decreased food intake [221]. Mc4r homozygous deletion leads to an obese phenotype in mice [222]. MC4R mutations in humans may induce obesity via haploinsufficiency, dominant-negative action, or a combination of the two resulting receptor functional changes. In addition to the decreased gene transcription caused by mutations in key areas of the MC4R promoter, obesity in people may also occur [221,223]. The proper functioning of MC4R protects against obesity [224]. Aminoglycoside and chaperones are utilized to treat the mutations of the MC4R protein to restore its normal functional activity [225,226]. Genetically modified null MC4R mice models exhibit obesity, insulin resistance, nonalcoholic steatohepatitis (NASH), nonalcoholic fatty liver disease (NAFLD), fibrosis, and hepatocellular carcinoma (HCC). Generally, different types of herbal products are utilized to treat these illnesses, such as Silibinin from milk thistle (*Silybum marianum*), lycopene from tomato, watermelon, papaya, orange, grapefruit, nobiletin from citrus fruit, baicalein from *Scutellaria baicalensis,* and quercetin from broccoli or onion [227].

### 7.4. Increase the Secretion of Adiponectin

Adiponectin is a collagen-like plasma protein with a molecular weight of 30 kDa that is produced by adipocytes but mainly circulates in hexameric, oligomeric, and, to a lesser degree, trimeric forms [228,229]. Adiponectin possesses insulin-mimetic and sensitizing measures, including glucose stimulation in the muscles and skeleton and glucose suppression in the liver. Adiponectin works with two receptor isoforms, AdipoR1 (adiponectin receptor 1) and AdipoR2, which have different tissues and related forms of adiponectin recognition [228]. Plasma adiponectin levels have a negative correlation with body fat percentage and waist-thickness ratio, while they have an inverse relationship with fasting plasma insulin levels [229]. The roots of the medicinal plant *Radix astragali* yield two types of saponins, astragaloside II and isoastragaloside I, which, when delivered to primary adipocytes, stimulate adiponectin secretion without impacting the release of other adipokines, offering a promising avenue for treating obesity-related illnesses [230].

## 8. Conclusions and Future Prospects

Phytogenic substances that alter obesity and diabetes are discussed in this review. While there has been some research toward a combined treatment for obesity and diabetes, there is currently no such treatment. Inflammation and insulin resistance are common features of both obesity and diabetes. As a result of the correlations between obesity and diabetes and the interesting properties of phytogenic synthetic substances, some phytogenic mixtures might be utilized to create medicines for the two issues. Herbal remedies, for instance, can assist with hyperglycemia and muscle versus fat synthesis. More examination into the potential outcomes of herbal products would be valuable. Plants can have perilous or inadequate impacts and unexplained results in regular item research, yet the choices are interminable. We characterize dynamic parts and normal items dependent on a lot of exploration and clinical preliminaries and clarify restorative plants with hostile to diabetic advantages in an extensive way, zeroing in on hormonal guidelines and metabolic guidelines.

## Figures and Tables

**Table 1:** Herbal therapeutics with their mode of action against diabetes.

Compound Name	Herbal Sources	Mode of Action	References
Kaempferol	Citrus, berry, grape, and soybean	Inhibition of DPP-4	[204]
Malvidin			
Epigallocatechin gallate			
Cyanidin-3-glucoside			
Gallic acid			
Luteolin			
Apigenin			
Quercetin			
Flavone			
Hesperetin			
Naringenin			
Eriocitrin			
Resveratrol			
Caffeic acid			
Cyanidin			
Genistein			
Isoquercitrin	Flowers of *Gossypium herbaceum* L. (Malvaceae) and leaves of *Apocynumcannabinum* L. (Apocynaceae)		
Naringenin	*Rosmarinus officinalis* L. (Labiatae) and greenhouse-grown Mexican *Lippia graveolens* Kunth (Labiatae)		
Eriodictyol			
Hispidulin			
Cirsimaritin			
Rosmarinic acid			
Carnosol			
Naringin	*Citrus aurantium* L. (Rutaceae) and Peels of *Citrus maxima* Merr.		
Berberine	Chinese herb *Coptis chinensis* French. (Ranunculaceae)		
Rebaudioside A	*Stevia rebaudiana* (Bertoni) Hemsl (Asteraceae)		
stevioside			
Curcumin	*Curcuma longa*	Inhibition of PTP1B	[14,205,206,207]
Cinnamaldehyde	Cinnamon trees		
ethyl acetate (EtOAc)	Methanolic extract of the root of *P. cuspidatum*		
Eicosenoic acid	Bark of *Phellodendronamurense* Rupr		
vaccenic acid			
oleic acid			
linoleic acid			
petroselinic acid			
palmitoleic acid			
palmitic acid	*Agrimonia pilosa*		
Vasicine	Methanolic extract of *Adhatoda vasica*	Inhibition of α-Glycosidase	[208]
Vasicinol			
Piperumbellactam A	Branches of *Piper umbellatum*		
PiperumbellactamB			
Piperumbellactam C			
3,4-dicaffeoylquinic acid	Methanolic extract from flower buds of *Tussilago farfara*		
4,5-dicaffeoylquinic acid			
Chebulanin	70% methanolic extract from dried *Terminalia chebula* (Combretaceae) fruits		
Chebulagic acid			
Chebulinic acid			
(-)-3-O-galloylepicatechin	50% methanolic extract from *Bergenia cilata*		
Curcumin	*Curcuma longa* (turmeric)		
Demethoxycurcumin			
Bisdemethoxycurcumin			
Resveratrol	Grapes and red wine	Activation of Nrf2	[208]
Pterostilbene	Blueberry		
Caffeic acid	Coffee		
Desoxyrhapontigenin	*Rheum undulatum* L.		
Oxyresveratrol	Mulberry		
Polydatin	*Polygonum cuspidatum*		
Caffeic acid phenethyl ester	Honeybee propolis		
Hydroxytyrosol acetate	Olive		
Hydroxytyrosol butyrate			
Epigallocatechin gallate (EGCG)	Green tea		
Hesperetin	*Aurantium*		
Isoliquiritin (ILQ)	*Glycyrrhiza*		
Isoliquiritigenin (ILG)			
Kinsenoside	*Anoectochilus roxburghii*	Modification of pancreatic beta-cell	[198]
Silymarin	*Silybum marianum*		
Berberine	*Rhizomacoptidis*		
Nymphayol	*Nymphaea stellate*		
Momordicin	*Momordica charantia*		
Genistein	*Glycine max*		
Conophylline	*Ervatamia microphylla*		
Curcumin	*Curcuma longa*		
Capsaicin	*Capsicum annuum*		
Epigallocatechin-3-gallate	*Camellia sinensis*		
Curcumin	*Curcuma longa* (Turmeric)	Inhibition of Aldose reductase enzyme	[209]
Ellagic acid	*Phyllanthus niruni* L. (Euphorbiaceae)		
Berberine	*Mahonia aquifolium* (Oregon grape), *Tinosporacordifolia*, *Coptis chinensis* (Chinese goldthread), *Berberis vulgaris* (European barberry), *Philodendron bipinnatifidum* (Phellodendron), *Coptistrifolia* (Goldthread), *Berberis aristata* (tree turmeric), *Cortex phellodendri*, *Cosciniumfenestratum*(Yellow vine), and *Hydrastis canadensis* (Goldenseal), *Coptis japonica* (Japanese goldthread)		
Quercetin	Tomato, red grapes, leafy green vegetables, broccoli, citrus fruit		
Maesanin	Fruits *of Maesa lanceolata* (Myrsinaceae)		
Brevifolin carboxylic acid	*Phyllanthus nirun*		
Dehydrocorydaline	Tuber of *Corydalis turstchaninovii*		
Flaviolin	Fruits of *Maesalanceolata* (Myrsinaceae)		
Salvianolic acid A	*Salvia miltiorhiza*		
Lithospermic acid B	Root of *Salvia deserta*		
Kotalagenin 16-acetate	Root of *Salacia oblonga* Wall (Celastraceae)		
Acteoside	*Monochasmasavatierii*, Plantagoasiatica		
Myrciaphenone B	*Myrcia multiflora* (Myrtaceae)		
Chlorogenic acid	*Chrysanthemunindicum* L. (Compositae)		
Gossypol	*Gossypium* Sp. (Malvaceae)		
Dibenzocyclooctane	*Schisandra chinensis*		
Brazilin	*Caesalphiniasappan* (Leguminosae)		
Haematoxylin	*Haematoxylum campechianum*		
Furoguaiaoxidin	Resin of *Guaiacum officinale* L.		
Resveratrol	Grapes, red wine, and peanuts	Regulation of autophagy	[185]
Berberine	Coptischinensis		
Quercetin	Vegetables, fruits, and teas		
Dihydromyricetin	*Ampelopsis grossedentata*		
Epigallocatechin gallate (EGCG)	Green tea		

**Table 2:** Herbal therapeutics with their mode of action against obesity.

Compound Name	Herbal Sources	Mode of Action	References
Lutein	Green tea leaves	Inhibition of Aryl hydrocarbon receptors	[85,216]
Chlorophyll a			
Chlorophyll b			
(-)-Epigallocatechin gallate			
Silymarin	Milk thistle (*Silybummarianum* SL)	Inhibition of adipogenesis by methylxanthine	[220]
Caffeine	*Coffeacanephora*, various tea brush, and yerba maté		
Curcumin	*Curcuma longa*		
*p*-synephrine	*Citrus aurantium*		
Resveratrol	Berries of the wine grape		
Silibinin	Milk thistle (*Silybummarianum*)	Recover the disruption of melanocortin 4 receptor (*MC4R*) protein	[227]
Lycopene	Tomato, watermelon, papaya, orange, grapefruit		
Nobiletin	Citrus fruit		
Baicalein	*Scutellariabaicalensis Georgi*		
Quercetin	Broccoli, onion		
Astragaloside II	*Radix astragali*	Increase the secretion of adiponectin	[230]
Isoastragaloside I			

## Data Availability

Available data are presented in the manuscript.

## References

[1] Liu Q., Chen L., Hu L., Guo Y., Shen X. (2010). Small molecules from natural sources, targeting signaling pathways in diabetes. Biochim. Biophys. Acta-Gene Regul. Mech..

[2] Hung H.Y., Qian K., Morris-Natschke S.L., Hsu C.S., Lee K.H. (2012). Recent discovery of plant-derived anti-diabetic natural products. Nat. Prod. Rep..

[3] Bhardwaj M., Yadav P., Vashishth D., Sharma K., Kumar A., Chahal J., Dalal S., Kataria S.K. (2021). A review on obesity management through natural compounds and a green nanomedicine-based approach. Molecules.

[4] Mohamed G.A., Ibrahim S.R.M., Elkhayat E.S., El Dine R.S. (2014). Natural anti-obesity agents. Bull. Fac. Pharm. Cairo Univ..

[5] Oh S., Kim K., Chung Y., Shong M., Park S. (2009). Anti-obesity Agents: A Focused Review on the Structural Classification of Therapeutic Entities. Curr. Top. Med. Chem..

[6] Hatware K.V., Sharma S., Patil K., Shete M., Karri S., Gupta G. (2018). Evidence for gastroprotective, anti-inflammatory and antioxidant potential of methanolic extract of *Cordia dichotoma* leaves on indomethacin and stress induced gastric lesions in Wistar rats. Biomed. Pharmacother..

[7] Das R., Mitra S., Tareq A.M., Emran T.B., Hossain M.J., Alqahtani A.M., Alghazwani Y., Dhama K., Simal-Gandara J. (2022). Medicinal plants used against hepatic disorders in Bangladesh: A comprehensive review. J. Ethnopharmacol..

[8] Sun N.N., Wu T.Y., Chau C.F. (2016). Natural dietary and herbal products in anti-obesity treatment. Molecules.

[9] Karri S., Sharma S., Hatware K., Patil K. (2019). Natural anti-obesity agents and their therapeutic role in management of obesity: A future trend perspective. Biomed. Pharmacother..

[10] Lahlou M. (2013). The Success of Natural Products in Drug Discovery. Pharmacol. Pharm..

[11] Lam K.S. (2007). New aspects of natural products in drug discovery. Trends Microbiol..

[12] Kingston D.G.I. (2011). Modern natural products drug discovery and its relevance to biodiversity conservation. J. Nat. Prod..

[13] Mishra B.B., Tiwari V.K. (2011). Natural products: An evolving role in future drug discovery. Eur. J. Med. Chem..

[14] Kostrzewa T., Przychodzen P., Gorska-Ponikowska M., Kuban-Jankowska A. (2019). Curcumin and cinnamaldehyde as PTP1B inhibitors with antidiabetic and anticancer potential. Anticancer Res..

[15] Dias D.A., Urban S., Roessner U. (2012). A Historical overview of natural products in drug discovery. Metabolites.

[16] Zaid H., Saad B. (2013). State of the Art of Diabetes Treatment in Greco-Arab and Islamic Medicine. Bioact. Food Diet. Interv. Diabetes.

[17] Zaid H., Saad B., Mahdi A.A., Tamrakar A.K., Haddad P.S., Afifi F.U. (2015). Medicinal Plants and Natural Active Compounds for Diabetes and/or Obesity Treatment. Evid.-Based Complement. Altern. Med..

[18] (2007). Pankaj Modi Diabetes Beyond Insulin: Review of New Drugs for Treatment of Diabetes Mellitus. Curr. Drug Discov. Technol..

[19] Rahman M.M., Islam M.R., Islam M.T., Harun-Or-rashid M., Islam M., Abdullah S., Uddin M.B., Das S., Rahaman M.S., Ahmed M. (2022). Stem Cell Transplantation Therapy and Neurological Disorders: Current Status and Future Perspectives. Biology.

[20] Neustadt J., Pieczenik S.R. (2008). Medication-induced mitochondrial damage and disease. Mol. Nutr. Food Res..

[21] Cao Y., Liu X.M. (2015). Should we still be concerned about the potential side effects of glucagon-like peptide-1 receptor agonists on thyroid C cells?. Endocrine.

[22] Patel D.K., Prasad S.K., Kumar R., Hemalatha S. (2012). An overview on antidiabetic medicinal plants having insulin mimetic property. Asian Pac. J. Trop. Biomed..

[23] Arulselvan P., Ghofar H.A.A., Karthivashan G., Halim M.F.A., Ghafar M.S.A., Fakurazi S. (2014). Antidiabetic therapeutics from natural source: A systematic review. Biomed. Prev. Nutr..

[24] Gothai S., Ganesan P., Park S.Y., Fakurazi S., Choi D.K., Arulselvan P. (2016). Natural phyto-bioactive compounds for the treatment of type 2 diabetes: Inflammation as a target. Nutrients.

[25] Redinger R.N. (2007). The pathophysiology of obesity and its clinical manifestations. Gastroenterol. Hepatol..

[26] Nagaraju G.P., Aliya S., Alese O.B. (2015). Role of adiponectin in obesity related gastrointestinal carcinogenesis. Cytokine Growth Factor Rev..

[27] Chung S.J., Nagaraju G.P., Nagalingam A., Muniraj N., Kuppusamy P., Walker A., Woo J., Győrffy B., Gabrielson E., Saxena N.K. (2017). ADIPOQ/adiponectin induces cytotoxic autophagy in breast cancer cells through STK11/LKB1-mediated activation of the AMPK-ULK1 axis. Autophagy.

[28] Muppala S., Konduru S.K.P., Merchant N., Ramsoondar J., Rampersad C.K., Rajitha B., Mukund V., Kancherla J., Hammond A., Barik T.K. (2017). Adiponectin: Its role in obesity-associated colon and prostate cancers. Crit. Rev. Oncol. Hematol..

[29] Deng Z.B., Liu Y., Liu C., Xiang X., Wang J., Cheng Z., Shah S.V., Zhang S., Zhang L., Zhuang X. (2009). Immature myeloid cells induced by a high-fat diet contribute to liver inflammation. Hepatology.

[30] Hossain P., Kawar B., El Nahas M. (2007). Obesity and Diabetes in the Developing World—A Growing Challenge. N. Engl. J. Med..

[31] Jung H.S., Lim Y., Kim E.K. (2014). Therapeutic phytogenic compounds for obesity and diabetes. Int. J. Mol. Sci..

[32] Jebb S. (2004). Obesity: Causes and consequences. Women’s Health Med..

[33] Kopelman P.G. (2000). Obesity as a medical problem. Nature.

[34] Jahan I., Tona M.R., Sharmin S., Sayeed M.A., Tania F.Z., Paul A., Chy M., Uddin N., Rakib A., Emran T.B. (2020). GC-MS phytochemical profiling, pharmacological properties, and in silico studies of *Chukrasia velutina* leaves: A novel source for bioactive agents. Molecules.

[35] Friedman J.M. (2009). Obesity: Causes and control of excess body fat. Nature.

[36] Näslund E., Hellström P.M. (2007). Appetite signaling: From gut peptides and enteric nerves to brain. Physiol. Behav..

[37] Dutta T., Paul A., Majumder M., Sultan R.A., Emran T.B. (2020). Pharmacological evidence for the use of *Cissus assamica* as a medicinal plant in the management of pain and pyrexia. Biochem. Biophys. Rep..

[38] Padwal R.S., Majumdar S.R. (2007). Drug treatments for obesity: Orlistat, sibutramine, and rimonabant. Lancet.

[39] Association A.D. (2004). Diagnosis and Classification of Diabetes Mellitus. Diabetes Care.

[40] Chakraborty A.J., Uddin T.M., Zidan M., Redwan B.M., Mitra S., Das R., Nainu F., Dhama K., Roy A., Hossain M. (2022). *Allium cepa*: A Treasure of Bioactive Phytochemicals with Prospective Health Benefits. Evid. Based Complement. Altern. Med..

[41] Ripsin C.M., Kang H., Urban R.J. (2009). Management of blood glucose in type 2 diabetes mellitus. Am. Fam. Physician.

[42] Men P., Qu S., Song Z., Liu Y., Li C., Zhai S. (2020). Lixisenatide for Type 2 Diabetes Mellitus Patients Inadequately Controlled on Oral Antidiabetic Drugs: A Mixed-Treatment Comparison Meta-analysis and Cost–Utility Analysis. Diabetes Ther..

[43] Tirla A., Vesa C.M., Cavalu S. (2021). Severe Cardiac and Metabolic Pathology Induced by Steroid Abuse in a Young Individual. Diagnostics.

[44] Xu L., Li Y., Dai Y., Peng J. (2018). Natural products for the treatment of type 2 diabetes mellitus: Pharmacology and mechanisms. Pharmacol. Res..

[45] Oguma Y., Sesso H.D., Paffenbarger R.S., Lee I.M. (2005). Weight change and risk of developing type 2 diabetes. Obes. Res..

[46] Wannamethee S.G., Shaper A.G., Walker M. (2005). Overweight and obesity and weight change in middle aged men: Impact on cardiovascular disease and diabetes. J. Epidemiol. Community Health.

[47] Nagaya T., Yoshida H., Takahashi H., Kawai M. (2005). Increases in body mass index, even within non-obese levels, raise the risk for Type 2 diabetes mellitus: A follow-up study in a Japanese population. Diabet. Med..

[48] Meigs J.B., Wilson P.W.F., Fox C.S., Vasan R.S., Nathan D.M., Sullivan L.M., D’Agostino R.B. (2006). Body mass index, metabolic syndrome, and risk of type 2 diabetes or cardiovascular disease. J. Clin. Endocrinol. Metab..

[49] Antonescu A.-I., Miere F., Fritea L., Ganea M., Zdrinca M., Dobjanschi L., Antonescu A., Vicas S.I., Bodog F., Sindhu R.K. (2021). Perspectives on the Combined Effects of *Ocimum basilicum* and *Trifolium pratense* Extracts in Terms of Phytochemical Profile and Pharmacological Effects. Plants.

[50] Weyer C., Funahashi T., Tanaka S., Hotta K., Matsuzawa Y., Pratley R.E., Tataranni P.A. (2001). Hypoadiponectinemia in obesity and type 2 diabetes: Close association with insulin resistance and hyperinsulinemia. J. Clin. Endocrinol. Metab..

[51] De Ferranti S., Mozaffarian D. (2008). The perfect storm: Obesity, adipocyte dysfunction, and metabolic consequences. Clin. Chem..

[52] Deng Y., Scherer P.E. (2010). Adipokines as novel biomarkers and regulators of the metabolic syndrome. Ann. N. Y. Acad. Sci..

[53] Pandya H., Lakhani J.D., Patel N. (2011). Obesity is becoming synonym for diabetes in rural areas of India also—An alarming situation. Int. J. Biol. Med. Res..

[54] Snehalatha C., Viswanathan V., Ramachandran A. (2003). Cutoff values for normal anthropometric variables in Asian Indian adults. Diabetes Care.

[55] The N.S., Richardson A.S., Gordon-Larsen P. (2013). Timing and duration of obesity in relation to diabetes: Findings from an ethnically diverse, nationally representative sample. Diabetes Care.

[56] Wormser D., Kaptoge S., Di Angelantonio E., Wood A.M., Pennells L., Thompson A., Sarwar N., Kizer J.R., Lawlor D.A., Nordestgaard B.G. (2011). Separate and combined associations of body-mass index and abdominal adiposity with cardiovascular disease: Collaborative analysis of 58 prospective studies. Lancet.

[57] Lindgren C.M., Heid I.M., Randall J.C., Lamina C., Steinthorsdottir V., Qi L., Speliotes E.K., Thorleifsson G., Willer C.J., Herrera B.M. (2009). Genome-wide association scan meta-analysis identifies three loci influencing adiposity and fat distribution. PLoS Genet..

[58] Scherag A., Dina C., Hinney A., Vatin V., Scherag S., Vogel C.I.G., Müller T.D., Grallert H., Wichmann H.E., Balkau B. (2010). Two new loci for body-weight regulation identified in a joint analysis of genome-wide association studies for early-onset extreme obesity in French and German study groups. PLoS Genet..

[59] Hayes M.G., Pluzhnikov A., Miyake K., Sun Y., Ng M.C.Y., Roe C.A., Below J.E., Nicolae R.I., Konkashbaev A., Bell G.I. (2007). Identification of type 2 diabetes genes in Mexican Americans through genome-wide association studies. Diabetes.

[60] Rampersaud E., Damcott C.M., Fu M., Shen H., McArdle P., Shi X., Shelton J., Yin J., Chang Y.P.C., Ott S.H. (2007). Identification of novel candidate genes for type 2 diabetes from a genome-wide association scan in the old order amish: Evidence for replication from diabetes-related quantitative traits and from independent populations. Diabetes.

[61] Lander E.S. (2011). Initial impact of the sequencing of the human genome. Nature.

[62] Bogardus C. (2009). Missing heritability and GWAS utility. Obesity.

[63] Loos R.J.F., Bouchard C. (2003). Obesity—Is it a genetic disorder?. J. Intern. Med..

[64] Elbers C.C., Onland-Moret N.C., Franke L., Niehoff A.G., van der Schouw Y.T., Wijmenga C. (2007). A strategy to search for common obesity and type 2 diabetes genes. Trends Endocrinol. Metab..

[65] Hu H., Li X. (2008). Networking pathways unveils association between obesity and non-insulin dependent diabetes mellitus. Pacific Symp. Biocomput..

[66] Kim J.H., Joung H.Y., Kang S.A., Pyun K.H., Shim I. (2007). Ginsenoside Rb1 as a suppressor in central modulation of feeding in the rat. Appetite.

[67] Xie J.T., Zhou Y.P., Dey L., Attele A.S., Wu J.A., Gu M., Polonsky K.S., Yuan C.S. (2002). Ginseng berry reduces blood glucose and body weight in db/db mice. Phytomedicine.

[68] Attele A.S., Zhou Y.P., Xie J.T., Wu J.A., Zhang L., Dey L., Pugh W., Rue P.A., Polonsky K.S., Yuan C.S. (2002). Antidiabetic effects of Panax ginseng berry extract and the identification of an effective component. Diabetes.

[69] MacLean D.B., Luo L.G. (2004). Increased ATP content/production in the hypothalamus may be a signal for energy-sensing of satiety: Studies of the anorectic mechanism of a plant steroidal glycoside. Brain Res..

[70] van Heerden F.R., Marthinus Horak R., Maharaj V.J., Vleggaar R., Senabe J.V., Gunning P.J. (2007). An appetite suppressant from Hoodia species. Phytochemistry.

[71] Jain S., Singh S.N. (2013). Metabolic effect of short term administration of Hoodia gordonii, an herbal appetite suppressant. S. Afr. J. Bot..

[72] Rahman M., Uddin M., Reza A.S.M., Tareq A.M., Emran T.B., Simal-Gandara J. (2021). Ethnomedicinal value of antidiabetic plants in Bangladesh: A comprehensive review. Plants.

[73] Vuong T., Benhaddou-Andaloussi A., Brault A., Harbilas D., Martineau L.C., Vallerand D., Ramassamy C., Matar C., Haddad P.S. (2009). Antiobesity and antidiabetic effects of biotransformed blueberry juice in KKA y mice. Int. J. Obes..

[74] Grace M.H., Ribnicky D.M., Kuhn P., Poulev A., Logendra S., Yousef G.G., Raskin I., Lila M.A. (2009). Hypoglycemic activity of a novel anthocyanin-rich formulation from lowbush blueberry, Vaccinium angustifolium Aiton. Phytomedicine.

[75] Basu A., Lyons T.J. (2012). Strawberries, blueberries, and cranberries in the metabolic syndrome: Clinical perspectives. J. Agric. Food Chem..

[76] Molan A.L., Lila M.A., Mawson J. (2008). Satiety in rats following blueberry extract consumption induced by appetite-suppressing mechanisms unrelated to in vitro or in vivo antioxidant capacity. Food Chem..

[77] Prior R.L., Wilkes S.E., Rogers T.R., Khanal R.C., Wu X., Howard L.R. (2010). Purified blueberry anthocyanins and blueberry juice alter development of obesity in mice fed an obesogenic high-fat diet. J. Agric. Food Chem..

[78] Prior R.L., Wu X., Gu L., Hager T.J., Hager A., Howard L.R. (2008). Whole berries versus berry anthocyanins: Interactions with dietary fat levels in the C57BL/6J mouse model of obesity. J. Agric. Food Chem..

[79] Yun J.W. (2010). Possible anti-obesity therapeutics from nature—A review. Phytochemistry.

[80] Azzu V., Jastroch M., Divakaruni A.S., Brand M.D. (2010). The regulation and turnover of mitochondrial uncoupling proteins. Biochim. Biophys. Acta-Bioenerg..

[81] Sridhar K.R., Bhat R. (2007). Lotus—A potential nutraceutical source. J. Agric. Technol. Bhat J. Agric. Technol..

[82] Ono Y., Hattori E., Fukaya Y., Imai S., Ohizumi Y. (2006). Anti-obesity effect of Nelumbo nucifera leaves extract in mice and rats. J. Ethnopharmacol..

[83] Ahn J.H., Kim E.S., Lee C., Kim S., Cho S.H., Hwang B.Y., Lee M.K. (2013). Chemical constituents from Nelumbo nucifera leaves and their anti-obesity effects. Bioorg. Med. Chem. Lett..

[84] Rahman M.M., Rahaman M.S., Islam M.R., Hossain M.E., Mithi F.M., Ahmed M., Saldías M., Akkol E.K., Sobarzo-Sánchez E. (2021). Multifunctional Therapeutic Potential of Phytocomplexes and Natural Extracts for Antimicrobial Properties. Antibiotics.

[85] Mitra S., Rauf A., Tareq A.M., Jahan S., Emran T.B., Shahriar T.G., Dhama K., Alhumaydhi F.A., Aljohani A.S.M., Rebezov M. (2021). Potential health benefits of carotenoid lutein: An updated review. Food Chem. Toxicol..

[86] Liu S., Li D., Huang B., Chen Y., Lu X., Wang Y. (2013). Inhibition of pancreatic lipase, α-glucosidase, α-amylase, and hypolipidemic effects of the total flavonoids from Nelumbo nucifera leaves. J. Ethnopharmacol..

[87] Islam M.S., Choi H. (2008). Dietary red chilli (*Capsicum frutescens* L.) is insulinotropic rather than hypoglycemic in type 2 diabetes model of rats. Phyther. Res..

[88] Rousset S., Alves-Guerra M.C., Mozo J., Miroux B., Cassard-Doulcier A.M., Bouillaud F., Ricquier D. (2004). The Biology of Mitochondrial Uncoupling Proteins. Diabetes.

[89] Lee M.S., Kim C.T., Kim I.H., Kim Y. (2011). Effects of capsaicin on lipid catabolism in 3T3-L1 adipocytes. Phyther. Res..

[90] Ohnuki K., Niwa S., Maeda S., Inoue N., Yazawa S., Fushiki T. (2001). CH-19 Sweet, a Non-Pungent Cultivar of Red Pepper, Increased Body Temperature and Oxygen Consumption in Humans. Biosci. Biotechnol. Biochem..

[91] Yoneshiro T., Aita S., Kawai Y., Iwanaga T., Saito M. (2012). Nonpungent capsaicin analogs (capsinoids) increase energy expenditure through the activation of brown adipose tissue in humans. Am. J. Clin. Nutr..

[92] Janssens P.L.H.R., Hursel R., Martens E.A.P., Westerterp-Plantenga M.S. (2013). Acute Effects of Capsaicin on Energy Expenditure and Fat Oxidation in Negative Energy Balance. PLoS ONE.

[93] Balentine D.A., Wiseman S.A., Bouwens L.C.M. (1997). The chemistry of tea flavonoids. Crit. Rev. Food Sci. Nutr..

[94] Bose M., Lambert J.D., Ju J., Reuhl K.R., Shapses S.A., Yang C.S. (2008). The green tea polyphenol, (−)-epigallocatechin-3-gallate, inhibits obesity and metabolic syndrome in high-fat fed mice. FASEB J..

[95] Raederstorff D.G., Schlachter M.F., Elste V., Weber P. (2003). Effect of EGCG on lipid absorption and plasma lipid levels in rats. J. Nutr. Biochem..

[96] Hursel R., Viechtbauer W., Dulloo A.G., Tremblay A., Tappy L., Rumpler W., Westerterp-Plantenga M.S. (2011). The effects of catechin rich teas and caffeine on energy expenditure and fat oxidation: A meta-analysis. Obes. Rev..

[97] Vendrame S., Daugherty A., Kristo A.S., Klimis-Zacas D. (2014). Wild blueberry (Vaccinium angustifolium)-enriched diet improves dyslipidaemia and modulates the expression of genes related to lipid metabolism in obese Zucker rats. Br. J. Nutr..

[98] Sheng X., Zhang Y., Gong Z., Huang C., Zang Y.Q. (2008). Improved insulin resistance and lipid metabolism by cinnamon extract through activation of peroxisome proliferator-activated receptors. PPAR Res..

[99] Sartorius T., Peter A., Schulz N., Drescher A., Bergheim I., MacHann J., Schick F., Siegel-Axel D., Schürmann A., Weigert C. (2014). Cinnamon extract improves insulin sensitivity in the brain and lowers liver fat in mouse models of obesity. PLoS ONE.

[100] Ziegenfuss T.N., Hofheins J.E., Mendel R.W., Landis J., Anderson R.A. (2006). Effects of a Water-Soluble Cinnamon Extract on Body Composition and Features of the Metabolic Syndrome in Pre-Diabetic Men and Women. J. Int. Soc. Sports Nutr..

[101] Foster G.D., Wyatt H.R., Hill J.O., McGuckin B.G., Brill C., Mohammed B.S., Szapary P.O., Rader D.J., Edman J.S., Klein S. (2003). A Randomized Trial of a Low-Carbohydrate Diet for Obesity. N. Engl. J. Med..

[102] Zhong L., Furne J.K., Levitt M.D. (2006). An extract of black, green, and mulberry teas causes malabsorption of carbohydrate but not of triacylglycerol in healthy volunteers. Am. J. Clin. Nutr..

[103] Hanhineva K., Törrönen R., Bondia-Pons I., Pekkinen J., Kolehmainen M., Mykkänen H., Poutanen K. (2010). Impact of dietary polyphenols on carbohydrate metabolism. Int. J. Mol. Sci..

[104] Asano N., Yamashita T., Yasuda K., Ikeda K., Kizu H., Kameda Y., Kato A., Nash R.J., Lee H.S., Ryu K.S. (2001). Polyhydroxylated alkaloids isolated from mulberry trees (*Morus alba* L.) and silkworms (*Bombyx mori* L.). J. Agric. Food Chem..

[105] Juhel C., Armand M., Pafumi Y., Rosier C., Vandermander J., Lairon D. (2000). Green tea extract (AR25^®^) inhibits lipolysis of triglycerides in gastric and duodenal medium in vitro. J. Nutr. Biochem..

[106] Birari R.B., Bhutani K.K. (2007). Pancreatic lipase inhibitors from natural sources: Unexplored potential. Drug Discov. Today.

[107] Bhathena S.J., Velasquez M.T. (2002). Beneficial role of dietary phytoestrogens in obesity and diabetes. Am. J. Clin. Nutr..

[108] Ali A.A., Velasquez M.T., Hansen C.T., Mohamed A.I., Bhathena S.J. (2005). Modulation of carbohydrate metabolism and peptide hormones by soybean isoflavones and probiotics in obesity and diabetes. J. Nutr. Biochem..

[109] Purkins L., Love E.R., Eve M.D., Wooldridge C.L., Cowan C., Smart T.S., Johnson P.J., Rapeport W.G. (2004). The influence of diet upon liver function tests and serum lipids in healthy male volunteers resident in a Phase I unit. Br. J. Clin. Pharmacol..

[110] DeFuria J., Bennett G., Strissel K.J., Perfield J.W., Milbury P.E., Greenberg A.S., Obin M.S. (2009). Dietary blueberry attenuates whole-body insulin resistance in high fat-fed mice by reducing adipocyte death and its inflammatory sequelae. J. Nutr..

[111] Surmi B.K., Hasty A.H. (2008). Macrophage infiltration into adipose tissue: Initiation, propagation and remodeling. Future Lipidol..

[112] Mills C.D. (2012). M1 and M2 macrophages: Oracles of health and disease. Crit. Rev. Immunol..

[113] Seymour E.M., Tanone I.I., Urcuyo-Llanes D.E., Lewis S.K., Kirakosyan A., Kondoleon M.G., Kaufman P.B., Bolling S.F. (2011). Blueberry intake alters skeletal muscle and adipose tissue peroxisome proliferator-activated receptor activity and reduces insulin resistance in obese rats. J. Med. Food.

[114] Stull A.J., Cash K.C., Johnson W.D., Champagne C.M., Cefalu W.T. (2010). Bioactives in blueberries improve insulin sensitivity in obese, insulin-resistant men and women. J. Nutr..

[115] Weidner C., De Groot J.C., Prasad A., Freiwald A., Quedenau C., Kliem M., Witzke A., Kodelja V., Han C.T., Giegold S. (2012). Amorfrutins are potent antidiabetic dietary natural products. Proc. Natl. Acad. Sci. USA.

[116] Emran T.B., Dutta M., Uddin M.M.N., Nath A.K., Uddin M.Z. (2015). Antidiabetic potential of the leaf extract of *Centella asiatica* in alloxan induced diabetic rats. Jahangirnagar Univ. J. Biol. Sci..

[117] Moller D.E. (2001). New drug targets for type 2 diabetes and the metabolic syndrome. Nature.

[118] Kaplan F., al-Majali K., Betteridge D.J. (2001). PPARs, insulin resistance and type 2 diabetes. Eur. J. Cardiovasc. Prev. Rehabil..

[119] Basch E., Ulbricht C., Kuo G., Szapary P., Smith M. (2003). Therapeutic applications of fenugreek. Altern. Med. Rev..

[120] Zia T., Hasnain S.N., Hasan S.K. (2001). Evaluation of the oral hypoglycaemic effect of *Trigonella foenum-graecum* L. (methi) in normal mice. J. Ethnopharmacol..

[121] Tiran D. (2003). The use of fenugreek for breast feeding women. Complement. Ther. Nurs. Midwifery.

[122] Ota A., Ulrih N.P. (2017). An overview of herbal products and secondary metabolites used for management of type two diabetes. Front. Pharmacol..

[123] Uemura T., Hirai S., Mizoguchi N., Goto T., Lee J.Y., Taketani K., Nakano Y., Shono J., Hoshino S., Tsuge N. (2010). Diosgenin present in fenugreek improves glucose metabolism by promoting adipocyte differentiation and inhibiting inflammation in adipose tissues. Mol. Nutr. Food Res..

[124] Gupta A., Gupta R., Lal B. (2001). Effect of Trigonella foenum-graecum (Fenugreek) Seeds on Glycaemic Control and Insulin Resistance in Type 2 Diabetes Mellitus: A Double Blind Placebo Controlled Study. J. Assoc. Physicians India.

[125] Rahman J., Tareq A.M., Hossain M., Sakib S.A., Islam M.N., Ali M., Uddin A.B.M., Hoque M., Nasrin M., Emran T.B. (2020). Biological evaluation, DFT calculations and molecular docking studies on the antidepressant and cytotoxicity activities of *Cycas pectinata* Buch.-Ham. Compounds. Pharmaceuticals.

[126] Sharma R.D., Sarkar A., Hazra D.K., Mishra B., Singh J.B., Sharma S.K., Maheshwari B.B., Maheshwari P.K. (1996). Use of Fenugreek seed powder in the management of non-insulin dependent diabetes mellitus. Nutr. Res..

[127] Kabir M.S.H., Hossain M.M., Kabir M.I., Rahman M.M., Hasanat A., Emran T.B., Rahman M.A. (2016). Phytochemical screening, Antioxidant, Thrombolytic, alpha-amylase inhibition and cytotoxic activities of ethanol extract of *Steudnera colocasiifolia* K. Koch leaves. J. Young Pharm..

[128] Rafehi H., Ververis K., Karagiannis T.C. (2012). Controversies surrounding the clinical potential of cinnamon for the management of diabetes. Diabetes Obes. Metab..

[129] Qin B., Panickar K.S., Anderson R.A. (2010). Cinnamon: Potential role in the prevention of insulin resistance, metabolic syndrome, and type 2 diabetes. J. Diabetes Sci. Technol..

[130] Imparl-Radosevich J., Deas S., Polansky M.M., Baedke D.A., Ingebritsen T.S., Anderson R.A., Graves D.J. (1998). Regulation of PTP-1 and insulin receptor kinase by fractions from cinnamon: Implications for cinnamon regulation of insulin signalling. Horm. Res..

[131] Cao H., Polansky M.M., Anderson R.A. (2007). Cinnamon extract and polyphenols affect the expression of tristetraprolin, insulin receptor, and glucose transporter 4 in mouse 3T3-L1 adipocytes. Arch. Biochem. Biophys..

[132] Mitra S., Tareq A.M., Das R., Emran T.B., Nainu F., Chakraborty A.J., Ahmad I., Tallei T.E., Idris A.M., Simal-Gandara J. (2022). Polyphenols: A first evidence in the synergism and bioactivities. Food Rev. Int..

[133] Shanmugasundaram E.R.B., Rajeswari G., Baskaran K., Kumar B.R.R., Shanmugasundaram K.R., Ahmath B.K. (1990). Use of Gymnema sylvestre leaf extract in the control of blood glucose in insulin-dependent diabetes mellitus. J. Ethnopharmacol..

[134] Siddiqui A.A., Bahar A., Anshul D. (2000). Development in the chemistry and pharmacology of Gymnema sylvestre. J. Med. Aromat. Plant Sci..

[135] Tiwari A.K., Rao J.M. (2002). Diabetes mellitus and multiple therapeutic approaches of phytochemicals: Present status and future prospects. Curr. Sci..

[136] Baskaran K., Ahamath B.K., Shanmugasundaram K.R., Shanmugasundaram E.R.B. (1990). Antidiabetic effect of a leaf extract from Gymnema sylvestre in non-insulin-dependent diabetes mellitus patients. J. Ethnopharmacol..

[137] Balasubramaniam K., Seevaratnam S., Ageswaran A., Arasaratnam V., Thirumagal K. (1988). Hypoglycemic effect of Gymnema sylvestre on diabetic patients. Jaffna Med. J..

[138] Al Mahmud Z., Emran T.B., Qais N., Bachar S.C., Sarker M., Uddin M.M.N. (2016). Evaluation of analgesic, anti-inflammatory, thrombolytic and hepatoprotective activities of roots of *Premna esculenta* (Roxb). J. Basic Clin. Physiol. Pharmacol..

[139] Tiwari P., Mishra B.N., Sangwan N.S. (2014). Phytochemical and pharmacological properties of Gymnema sylvestre: An important medicinal plant. Biomed. Res. Int..

[140] Fujii M., Takei I., Umezawa K. (2009). Antidiabetic effect of orally administered conophylline-containing plant extract on streptozotocin-treated and Goto-Kakizaki rats. Biomed. Pharmacother..

[141] Kawakami M., Hirayama A., Tsuchiya K., Ohgawara H., Nakamura M., Umezawa K. (2010). Promotion of β-cell differentiation by the alkaloid conophylline in porcine pancreatic endocrine cells. Biomed. Pharmacother..

[142] Saito R., Yamada S., Yamamoto Y., Kodera T., Hara A., Tanaka Y., Kimura F., Takei I., Umezawa K., Kojima I. (2012). Conophylline suppresses pancreatic stellate cells and improves islet fibrosis in Goto-Kakizaki rats. Endocrinology.

[143] Zhang Y., Cai J., Ruan H., Pi H., Wu J. (2007). Antihyperglycemic activity of kinsenoside, a high yielding constituent from Anoectochilus roxburghii in streptozotocin diabetic rats. J. Ethnopharmacol..

[144] Subash-Babu P., Ignacimuthu S., Agastian P., Varghese B. (2009). Partial regeneration of β-cells in the islets of Langerhans by Nymphayol a sterol isolated from *Nymphaea stellata* (Willd.) flowers. Bioorg. Med. Chem..

[145] Diabetes C.O.F., Management D. (1998). Herbal Support for Diabetes Management. Clin. Nutr. Insights.

[146] Saxena A., Vikram N.K. (2004). Role of Selected Indian Plants in Management of Type 2 Diabetes: A Review. J. Altern. Complement. Med..

[147] Grover J.K., Yadav S., Vats V. (2002). Medicinal plants of India with anti-diabetic potential. J. Ethnopharmacol..

[148] Das R., Lami M.S., Chakraborty A.J., Mitra S., Tallei T.E., Idroes R., Mohamed A.A.-R., Hossain M.J., Dhama K., Noor-E-Tabassum (2022). Ginkgo biloba: A Treasure of Functional Phytochemicals with Multimedicinal Applications. Evid. Based Complement. Altern. Med..

[149] Bhatt J.K., Thomas S., Nanjan M.J. (2012). Resveratrol supplementation improves glycemic control in type 2 diabetes mellitus. Nutr. Res..

[150] Szkudelska K., Szkudelski T. (2010). Resveratrol, obesity and diabetes. Eur. J. Pharmacol..

[151] Mitra S., Paul S., Roy S., Sutradhar H., Emran T.B., Nainu F., Khandaker M.U., Almalki M., Wilairatana P., Mubarak M.S. (2022). Exploring the Immune-Boosting Functions of Vitamins and Minerals as Nutritional Food Bioactive Compounds: A Comprehensive Review. Molecules.

[152] Aribal-Kocatürk P., Özelçi Kavas G., İren Büyükkağnici D. (2007). Pretreatment effect of resveratrol on streptozotocin-induced diabetes in rats. Biol. Trace Elem. Res..

[153] Ramadori G., Gautron L., Fujikawa T., Vianna C.R., Elmquist J.K., Coppari R. (2009). Central administration of resveratrol improves diet-induced diabetes. Endocrinology.

[154] Ungvari Z., Csiszar A. (2011). Resveratrol Confers Endothelial Protection in Insulin-Dependent Diabetes Mellitus. Cardiovasc. Drugs Ther..

[155] Zhang H., Morgan B., Potter B.J., Ma L., Dellsperger K.C., Ungvari Z., Zhang C. (2010). Resveratrol improves left ventricular diastolic relaxation in type 2 diabetes by inhibiting oxidative/nitrative stress: In vivo demonstration with magnetic resonance imaging. Am. J. Physiol.-Heart Circ. Physiol..

[156] Resmi H. (2011). The combination of bortezomib and resveratrol may prevent muscle wasting in diabetes. Med. Hypotheses.

[157] Hong Y.J., Kim N., Lee K., Hee Sonn C., Eun Lee J., Tae Kim S., Ho Baeg I., Lee K.M. (2012). Korean red ginseng (Panax ginseng) ameliorates type 1 diabetes and restores immune cell compartments. J. Ethnopharmacol..

[158] Huang J.P., Huang S.S., Deng J.Y., Chang C.C., Day Y.J., Hung L.M. (2010). Insulin and resveratrol act synergistically, preventing cardiac dysfunction in diabetes, but the advantage of resveratrol in diabetics with acute heart attack is antagonized by insulin. Free Radic. Biol. Med..

[159] Venturini C.D., Merlo S., Souto A.A., Fernandes M.D.C., Gomez R., Rhoden C.R. (2010). Resveratrol and red wine function as antioxidants in the central nervous system without cellular proliferative effects during experimental diabetes. Oxid. Med. Cell. Longev..

[160] Do G.M., Jung U.J., Park H.J., Kwon E.Y., Jeon S.M., Mcgregor R.A., Choi M.S. (2012). Resveratrol ameliorates diabetes-related metabolic changes via activation of AMP-activated protein kinase and its downstream targets in db/db mice. Mol. Nutr. Food Res..

[161] Ding D.F., You N., Wu X.M., Xu J.R., Hu A.P., Ye X.L., Zhu Q., Jiang X.Q., Miao H., Liu C. (2010). Resveratrol attenuates renal hypertrophy in early-stage diabetes by activating AMPK. Am. J. Nephrol..

[162] Mishra R., Sellin D., Radovan D., Gohlke A., Winter R. (2009). Inhibiting islet amyloid polypeptide fibril formation by the red wine compound resveratrol. ChemBioChem.

[163] Ku C.R., Lee H.J., Kim S.K., Lee E.Y., Lee M.K., Lee E.J. (2012). Resveratrol prevents streptozotocin-induced diabetes by inhibiting the apoptosis of pancreatic β-cell and the cleavage of poly(ADP-ribose) polymerase. Endocr. J..

[164] Vetterli L., Brun T., Giovannoni L., Bosco D., Maechler P. (2011). Resveratrol potentiates glucose-stimulated insulin secretion in INS-1E β-cells and human islets through a SIRT1-dependent mechanism. J. Biol. Chem..

[165] Haigis M.C., Sinclair D.A. (2010). Mammalian sirtuins: Biological insights and disease relevance. Annu. Rev. Pathol. Mech. Dis..

[166] Pacholec M., Bleasdale J.E., Chrunyk B., Cunningham D., Flynn D., Garofalo R.S., Griffith D., Griffor M., Loulakis P., Pabst B. (2010). SRT1720, SRT2183, SRT1460, and resveratrol are not direct activators of SIRT1. J. Biol. Chem..

[167] Kantsadi A.L., Apostolou A., Theofanous S., Stravodimos G.A., Kyriakis E., Gorgogietas V.A., Chatzileontiadou D.S.M., Pegiou K., Skamnaki V.T., Stagos D. (2014). Biochemical and biological assessment of the inhibitory potency of extracts from vinification byproducts of Vitis vinifera extracts against glycogen phosphorylase. Food Chem. Toxicol..

[168] Aoki F., Honda S., Kishida H., Kitano M., Arai N., Tanaka H., Yokota S., Nakagawa K., Asakura T., Nakai Y. (2007). Suppression by licorice flavonoids of abdominal fat accumulation and body weight gain in high-fat diet-induced obese C57BL/6J mice. Biosci. Biotechnol. Biochem..

[169] Kim H.K., Nelson-Dooley C., Della-Fera M.A., Yang J.Y., Zhang W., Duan J., Hartzell D.L., Hamrick M.W., Baile C.A. (2006). Genistein decreases food intake, body weight, and fat pad weight and causes adipose tissue apoptosis in ovariectomized female mice. J. Nutr..

[170] Naaz A., Yellayi S., Zakroczymski M.A., Bunick D., Doerge D.R., Lubahn D.B., Helferich W.G., Cooke P.S. (2003). The soy isoflavone genistein decreases adipose deposition in mice. Endocrinology.

[171] Brennan A.M., Mantzoros C.S. (2006). Drug Insight: The role of leptin in human physiology and pathophysiology—Emerging clinical applications. Nat. Clin. Pract. Endocrinol. Metab..

[172] Ae Park S., Choi M.S., Cho S.Y., Seo J.S., Jung U.J., Kim M.J., Sung M.K., Park Y.B., Lee M.K. (2006). Genistein and daidzein modulate hepatic glucose and lipid regulating enzyme activities in C57BL/KsJ-db/db mice. Life Sci..

[173] Choi M.S., Jung U.J., Yeo J., Kim M.J., Lee M.K. (2008). Genistein and daidzein prevent diabetes onset by elevating insulin level and altering hepatic gluconeogenic and lipogenic enzyme activities in non-obese diabetic (NOD) mice. Diabetes. Metab. Res. Rev..

[174] Liu D., Zhen W., Yang Z., Carter J.D., Si H., Reynolds K.A. (2006). Genistein acutely stimulates insulin secretion in pancreatic β-cells through a cAMP-dependent protein kinase pathway. Diabetes.

[175] Fu Z., Liu D. (2009). Long-term exposure to genistein improves insulin secretory function of pancreatic β-cells. Eur. J. Pharmacol..

[176] Oetjen E. (2013). Amorfrutins are potent antidiabetic dietary natural products. Yearb. Endocrinol..

[177] Kamisoyama H., Honda K., Tominaga Y., Yokota S., Hasegawa S. (2008). Investigation of the anti-obesity action of licorice flavonoid oil in diet-induced obese rats. Biosci. Biotechnol. Biochem..

[178] Tominaga Y., Nakagawa K., Mae T., Kitano M., Yokota S., Arai T., Ikematsu H., Inoue S. (2009). Licorice flavonoid oil reduces total body fat and visceral fat in overweight subjects: A randomized, double-blind, placebo-controlled study. Obes. Res. Clin. Pract..

[179] Mominur Rahman M., Islam F., Saidur Rahaman M., Sultana N.A., Fahim N.F., Ahmed M. (2021). Studies on the prevalence of HIV/AIDS in Bangladesh including other developing countries. Adv. Tradit. Med..

[180] Saito M., Yoneshiro T. (2013). Capsinoids and related food ingredients activating brown fat thermogenesis and reducing body fat in humans. Curr. Opin. Lipidol..

[181] Gram D.X., Ahrén B., Nagy I., Olsen U.B., Brand C.L., Sundler F., Tabanera R., Svendsen O., Carr R.D., Santha P. (2007). Capsaicin-sensitive sensory fibers in the islets of Langerhans contribute to defective insulin secretion in Zucker diabetic rat, an animal model for some aspects of human type 2 diabetes. Eur. J. Neurosci..

[182] Grover J.K., Vats V., Rathi S.S., Dawar R. (2001). Traditional Indian anti-diabetic plants attenuate progression of renal damage in streptozotocin induced diabetic mice. J. Ethnopharmacol..

[183] Huang H.L., Hong Y.W., Wong Y.H., Chen Y.N., Chyuan J.H., Huang C.J., Chao P. (2008). min Bitter melon (*Momordica charantia* L.) inhibits adipocyte hypertrophy and down regulates lipogenic gene expression in adipose tissue of diet-induced obese rats. Br. J. Nutr..

[184] Kar S., Roy K. (2012). QSAR of phytochemicals for the design of better drugs. Expert Opin. Drug Discov..

[185] Zhang X.W., Zhou J.C., Hu Z.W. (2017). Autophagy as a target for development of anti-diabetes drugs derived from natural compounds. J. Asian Nat. Prod. Res..

[186] Wais M., Nazish I., Samad A., Beg S., Abusufyan S., Ajaz Ajaj S., Aqil M. (2012). Herbal Drugs for Diabetic Treatment: An Updated Review of Patents. Recent Pat. Antiinfect. Drug Discov..

[187] Lin S.R., Chang C.H., Tsai M.J., Cheng H., Chen J.C., Leong M.K., Weng C.F. (2019). The perceptions of natural compounds against dipeptidyl peptidase 4 in diabetes: From in silico to in vivo. Ther. Adv. Chronic Dis..

[188] Omar B., Ahrén B. (2014). Pleiotropic mechanisms for the glucose-lowering action of DPP-4 inhibitors. Diabetes.

[189] Da Silva Júnior W.S., De Godoy-Matos A.F., Kraemer-Aguiar L.G. (2015). Dipeptidyl peptidase 4: A new link between diabetes mellitus and atherosclerosis?. Biomed. Res. Int..

[190] Hussain H., Green I.R., Abbas G., Adekenov S.M., Hussain W., Ali I. (2019). Protein tyrosine phosphatase 1B (PTP1B) inhibitors as potential anti-diabetes agents: Patent review (2015–2018). Expert Opin. Ther. Pat..

[191] Banu S., Bhowmick A. (2017). Therapeutic Targets of Type 2 Diabetes: An Overview. MOJ Drug Des. Dev. Ther..

[192] Kumar G.S., Page R., Peti W. (2020). The mode of action of the Protein tyrosine phosphatase 1B inhibitor Ertiprotafib. PLoS ONE.

[193] Tomasik P., Horton D. (2012). Enzymatic conversions of starch. Adv. Carbohydr. Chem. Biochem..

[194] Türkan F., Taslimi P., Saltan F.Z. (2019). Tannic acid as a natural antioxidant compound: Discovery of a potent metabolic enzyme inhibitor for a new therapeutic approach in diabetes and Alzheimer’s disease. J. Biochem. Mol. Toxicol..

[195] Hossain U., Das A.K., Ghosh S., Sil P.C. (2020). An overview on the role of bioactive α-glucosidase inhibitors in ameliorating diabetic complications. Food Chem. Toxicol..

[196] Khursheed R., Singh S.K., Wadhwa S., Gulati M., Awasthi A. (2020). Therapeutic potential of mushrooms in diabetes mellitus: Role of polysaccharides. Int. J. Biol. Macromol..

[197] Reis A.A.d.S., Santos R.d.S., Cruz A.H.d.S., Silva E.G.d., Cruz A.D.d., Pedrino G.R. (2016). The Effect of Nrf2 on Diabetic Complications. A Master Regul. Oxidative Stress—The Transcription Factor Nrf2.

[198] Oh Y.S. (2015). Plant-derived compounds targeting pancreatic beta cells for the treatment of diabetes. Evid.-Based Complement. Altern. Med..

[199] Jiménez-Osorio A.S., González-Reyes S., Pedraza-Chaverri J. (2015). Natural Nrf2 activators in diabetes. Clin. Chim. Acta..

[200] Veeresham C., Rama Rao A., Asres K. (2014). Aldose reductase inhibitors of plant origin. Phyther. Res..

[201] Chen S., Khoury C., Ziyadeh F.N. (2013). Pathophysiology and Pathogenesis of Diabetic Nephropathy.

[202] Maitra S., Dutta D. (2020). Downregulation of Hexose Sugar Metabolism in Diabetes Decreases the Rate of Wound Healing.

[203] Patel D.K., Kumar R., Sairam K., Hemalatha S. (2012). Pharmacologically tested aldose reductase inhibitors isolated from plant sources—A concise report. Chin. J. Nat. Med..

[204] Gao Y., Zhu J., Li Z., Zhu W., Shi J., Jia Q., Li Y. (2015). Recent progress in natural products as DPP-4 inhibitors. Future Med. Chem..

[205] Le H.L., To D.C., Tran M.H., Do T.T., Nguyen P.H. (2020). Natural PTP1B Inhibitors From Polygonum cuspidatum and Their 2-NBDG Uptake Stimulation. Nat. Prod. Commun..

[206] Zhao B.T., Nguyen D.H., Le D.D., Choi J.S., Min B.S., Woo M.H. (2018). Protein tyrosine phosphatase 1B inhibitors from natural sources. Arch. Pharm. Res..

[207] Tagde P., Tagde P., Islam F., Tagde S., Shah M., Hussain Z.D., Rahman M.H., Najda A., Alanazi I.S., Germoush M.O. (2021). The multifaceted role of curcumin in advanced nanocurcumin form in the treatment and management of chronic disorders. Molecules.

[208] Kumar S., Narwal S., Kumar V., Prakash O. (2011). α-glucosidase inhibitors from plants: A natural approach to treat diabetes. Pharmacogn. Rev..

[209] Verma S.K., Thareja S. (2020). An Overview on Chemistry of Natural Aldose Reductase Inhibitors for the Management of Diabetic Complications.

[210] Chooi Y.C., Ding C., Magkos F. (2019). The epidemiology of obesity. Metabolism.

[211] Darbre P.D. (2017). Endocrine Disruptors and Obesity. Curr. Obes. Rep..

[212] Jaeger C., Tischkau S.A. (2016). Role of Aryl Hydrocarbon Receptor in Circadian Clock Disruption and Metabolic Dysfunction. Environ. Health Insights.

[213] McMillan B.J., Bradfield C.A. (2007). The aryl hydrocarbon receptor is activated by modified low-density lipoprotein. Proc. Natl. Acad. Sci. USA.

[214] Moyer B.J., Rojas I.Y., Kerley-Hamilton J.S., Hazlett H.F., Nemani K.V., Trask H.W., West R.J., Lupien L.E., Collins A.J., Ringelberg C.S. (2016). Inhibition of the aryl hydrocarbon receptor prevents Western diet-induced obesity. Model for AHR activation by kynurenine via oxidized-LDL, TLR2/4, TGFβ, and IDO1. Toxicol. Appl. Pharmacol..

[215] Kerley-Hamilton J.S., Trask H.W., Ridley C.J.A., Dufour E., Ringelberg C.S., Nurinova N., Wong D., Moodie K.L., Shipman S.L., Moore J.H. (2012). Obesity is mediated by differential aryl hydrocarbon receptor signaling in mice fed a western diet. Environ. Health Perspect..

[216] Fukuda I., Sakane I., Yabushita Y., Kodoi R., Nishiumi S., Kakuda T., Sawamura S.I., Kanazawa K., Ashida H. (2004). Pigments in Green Tea Leaves (*Camellia sinensis*) Suppress Transformation of the Aryl Hydrocarbon Receptor Induced by Dioxin. J. Agric. Food Chem..

[217] Moyer B.J., Rojas I.Y., Kerley-Hamilton J.S., Nemani K.V., Trask H.W., Ringelberg C.S., Gimi B., Demidenko E., Tomlinson C.R. (2017). Obesity and fatty liver are prevented by inhibition of the aryl hydrocarbon receptor in both female and male mice. Nutr. Res..

[218] Ali A.T., Hochfeld W.E., Myburgh R., Pepper M.S. (2013). Adipocyte and adipogenesis. Eur. J. Cell Biol..

[219] Kowalska K. (2011). Natural compounds involved in adipose tissue mass control in in vitro studies. Postepy Hig. Med. Dosw. (Online).

[220] Colitti M., Stefanon B. (2016). Different anti-adipogenic effects of bio-compounds on primary visceral pre-adipocytes and adipocytes. EXCLI J..

[221] Lubrano-Berthelier C., Cavazos M., Le Stunff C., Haas K., Shapiro A., Zhang S., Bougnerës P., Vaisse C. (2003). The Human MC4R Promoter: Characterization and Role in Obesity. Diabetes.

[222] Huszar D., Lynch C.A., Fairchild-Huntress V., Dunmore J.H., Fang Q., Berkemeier L.R., Gu W., Kesterson R.A., Boston B.A., Cone R.D. (1997). Targeted disruption of the melanocortin-4 receptor results in obesity in mice. Cell.

[223] Yeo G.S.H., Lank E.J., Farooqi I.S., Keogh J., Challis B.G., O’Rahilly S. (2003). Mutations in the human melanocortin-4 receptor gene associated with severe familial obesity disrupts receptor function through multiple molecular mechanisms. Hum. Mol. Genet..

[224] Lotta L.A., Mokrosiński J., Mendes de Oliveira E., Li C., Sharp S.J., Luan J., Brouwers B., Ayinampudi V., Bowker N., Kerrison N. (2019). Human Gain-of-Function MC4R Variants Show Signaling Bias and Protect against Obesity. Cell.

[225] Brumm H., Mühlhaus J., Bolze F., Scherag S., Hinney A., Hebebrand J., Wiegand S., Klingenspor M., Grüters A., Krude H. (2012). Rescue of melanocortin 4 receptor (MC4R) nonsense mutations by aminoglycoside-mediated read-through. Obesity.

[226] René P., Le Gouill C., Pogozheva I.D., Lee G., Mosberg H.I., Farooqi I.S., Valenzano K.J., Bouvier M. (2010). Pharmacological chaperones restore function to MC4R mutants responsible for severe early-onset obesity. J. Pharmacol. Exp. Ther..

[227] Sharma D., Saxena N.K. (2015). Mouse Models to Study the Effect of Natural Products on Obesity-Associated NAFLD/NASH. Murine Models, Energy Balance, and Cancer.

[228] Fang X., Sweeney G. (2006). Mechanisms regulating energy metabolism by adiponectin in obesity and diabetes. Biochem. Soc. Trans..

[229] Kawano J., Arora R. (2009). The Role of Adiponectin in Obesity, Diabetes, and Cardiovascular Disease. J. Cardiometab. Syndr..

[230] Xu A., Wang H., Hoo R.L.C., Sweeney G., Vanhoutte P.M., Wang Y., Wu D., Chu W., Qin G., Lam K.S.L. (2009). Selective Elevation of Adiponectin Production by the Natural Compounds Derived from a Medicinal Herb Alleviates Insulin Resistance and Glucose Intolerance in Obese Mice. Endocrinology.

